# Investigating potential transmission of antimicrobial resistance in an open-plan hospital ward: a cross-sectional metagenomic study of resistome dispersion in a lower middle-income setting

**DOI:** 10.1186/s13756-021-00915-w

**Published:** 2021-03-18

**Authors:** Anushia Ashokan, Josh Hanson, Ne Myo Aung, Mar Mar Kyi, Steven L. Taylor, Jocelyn M. Choo, Erin Flynn, Fredrick Mobegi, Morgyn S. Warner, Steve L. Wesselingh, Mark A. Boyd, Geraint B. Rogers

**Affiliations:** 1 Microbiome and Host Health, South Australia Health and Medical Research Institute, Adelaide, SA 5001 Australia; 2 SAHMRI Microbiome Research Laboratory, Flinders University College of Medicine and Public Health, Adelaide, SA Australia; 3 Faculty of Health and Medical Sciences, University of Adelaide, North Terrace, Adelaide, SA Australia; 4 Cairns Hospital, Cairns, QLD Australia; 5 Insein General Hospital, Insein, Yangon, Myanmar; 6 University of Medicine 2, Yangon, Myanmar; 7 South Australia (SA) Pathology, Adelaide, SA Australia; 8 South Australia Health and Medical Research Institute, Adelaide, SA Australia

**Keywords:** Antibiotic resistance, Metagenomics, Resource-limited settings, Healthcare, Resistome dispersion

## Abstract

**Background:**

Antimicrobial resistance (AMR) represents a profound global health threat. Reducing AMR spread requires the identification of transmission pathways. The extent to which hospital wards represent a venue for substantial AMR transmission in low- and middle-income countries settings is poorly understood.

**Methods:**

Rectal swabs were obtained from adult male inpatients in a “Nightingale” model general medicine ward in Yangon, Myanmar. Resistome characteristics were characterised by metagenomic sequencing. AMR gene carriage was related to inter-patient distance (representing inter-patient interaction) using distance-based linear models. Clinical predictors of AMR patterns were identified through univariate and multivariate regression.

**Results:**

Resistome similarity showed a weak but significant positive correlation with inter-patient distance (r = 0.12, *p* = 0.04). Nineteen AMR determinants contributed significantly to this relationship, including those encoding β-lactamase activity (*OXA-1*, *NDM-7*; adjusted *p* < 0.003), trimethoprim resistance (*dfrA14*, adjusted *p* = 0.0495), and chloramphenicol resistance (*catB3*, adjusted *p* = 0.002). Clinical traits of co-located patients carrying specific AMR genes were not random. Specifically, AMR genes that contributed to distance-resistome relationships (*OXA-1, catB3, dfrA14*) mapped to tuberculosis patients, who were placed together according to ward policy. In contrast, patients with sepsis were not placed together, and carried AMR genes that were not spatially significant or consistent with shared antibiotic exposure.

**Conclusions:**

AMR dispersion patterns primarily reflect the placement of particular patients by their condition, rather than AMR transmission. The proportion of AMR determinants that varied with inter-patient distance was limited, suggesting that nosocomial transmission is a relatively minor contributor to population-level carriage.

**Supplementary Information:**

The online version contains supplementary material available at 10.1186/s13756-021-00915-w.

## Background

Antimicrobial resistance (AMR) represents a profound health threat [1], particularly in low- and middle-income countries (LMIC) [2]. In many regions of South-East Asia, access to antibiotics is widespread but poorly regulated, and their use is often inappropriate [3, 4]. The off-prescription sale of antibiotics [3], and the unregulated use of critically important agents in animal husbandry [5, 6], is further compounded by high rates of infectious diseases, rapid unplanned urbanisation, poor sanitation [7], and inadequate waste management [8]. As a consequence, the World Health Organization (WHO) has identified South-East Asia as the global region at greatest risk from AMR [9].

The considerable challenges of addressing AMR are evident in Myanmar, a country of more than 53 million people. Following the end of military rule in 2011, Myanmar was reclassified in 2015 from a “low-income” to “lower-middle income” country (currently, 74th in global gross domestic product (GDP) rankings) [10]. However, despite growing national prosperity, a substantial portion of Myanmar’s population live in rural settings (69%), with many of those in urban settings dwelling in slums (41%) [11]. Expenditure on health remains low, representing 4·7% of GDP, substantially less than global averages (9·9%) [12]. Disability-adjusted life year (DALY), is high by global standards (394 per 1000 population, compared with a global average of 328) [13] and life expectancy remains below the global average (M/F: 64/69 versus 70/75) [14].

Myanmar released a National Action Plan for the Containment of Antimicrobial Resistance in 2017 [15], and in 2018, joined the WHO’s Global Antimicrobial Resistance Surveillance System [16]. Such commitments to coordinated surveillance are critically important. However, effective AMR containment is hampered by poor identification/description of the principal reservoirs of AMR and modes of AMR transmission.

Open plan hospital wards potentially represent an important venue for person-to-person AMR transmission. Despite single-bed hospital rooms becoming increasingly common in high-income countries (HICs), countries such as Myanmar continue to rely on large communal multi-bed “Nightingale” type wards for inpatient care where a combination of risk factors, including physical proximity, high antibiotic exposure, disruption of commensal microbiota, and increased bacterial dispersion [17], increase the likelihood of AMR dispersal.

Current AMR surveillance in hospitals typically employs culture-based approaches that focus on a narrow range of pathogens and on resistance to antibiotic agents that are of greatest clinical importance. Using such an approach, the potential to miss transmission events, which may be infrequent, involve non-target species, or relate to resistance to antibiotics not commonly used in the hospital setting is therefore high. Instead, understanding the process of ongoing AMR dispersal can be better achieved using an approach that captures all AMR genes within the patient microbiome, regardless of the bacterial species that carry them or the antibiotics to which they confer resistance. Shotgun metagenomic sequencing provides such a capacity, however, this genomic technology is usually unavailable in LMIC contexts.

Our aim was to better understand the extent of AMR transmission in an open plan hospital ward in a lower middle-income setting. We hypothesised that the occurrence of ongoing patient-to-patient AMR transmission within an open plan ward would be reflected in a greater similarity of resistome characteristics between patients, the greater their proximity.

## Methods

### Study population and sample collection

The study was undertaken in an adult male general medical ward at the Insein hospital, a 500-bed public tertiary teaching hospital in Yangon, Myanmar. Hospital infection control practices included the use of hand-sanitiser gel by staff prior to patient care, and the use of gloves and gowns for patients known to have transmissible infections or multi-resistant organisms. Bed rails were cleaned daily with a chlorhexidine-containing germicide (Septol), floors were cleaned daily with detergent, and other ward surfaces twice-weekly.

**Fig. 1:**
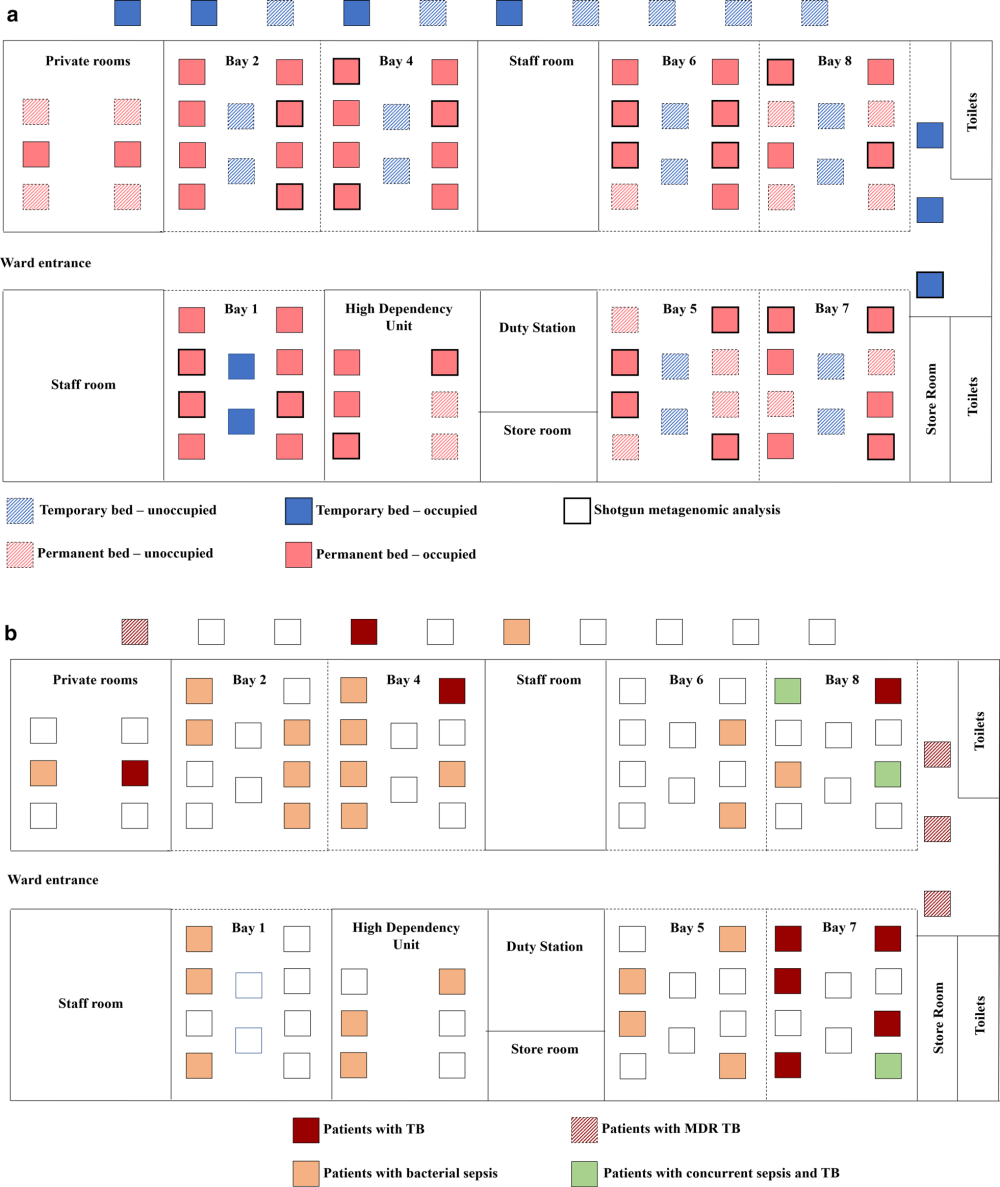
Ward map. Square denotes hospital bed where solid shading = patient occupied and pattern shading = unoccupied. Dotted lines represent bays separated by curtains and solid lines represent bays separated by walls. **a** location of study cohort and metagenomic sub-study **b** patients with sepsis (orange), tuberculosis (TB, maroon), multidrug resistant (MDR) TB (pattern shading, maroon) and sepsis + TB (green)

The hospital follows a policy of cohorting suspected and confirmed patients with TB (bays 7 & 8; Fig. 1). Two patients were listed as having TB but were located in bay 4 and the private room respectively. However, both are believed to have had extra-pulmonary TB. Where a patient is confirmed to have MDR-TB, they are moved to beds in the corridor prior to transfer to a dedicated TB hospital. N95 masks are used by medical staff for the routine care of all TB patients. Patients with TB were required to wear surgical masks. There were no dedicated negative pressure rooms in the hospital.

All included patients were provided with study information and consented to participate. Rectal swabs were collected on 19th January 2018 by medical professionals in accordance with hospital guidelines, frozen, and transported on dry ice to the South Australian Health and Medical Research Institute (SAHMRI), Australia where it was stored at -80℃ until analysis. Ethics approval for the study was received from Review Committee 1, University of Medicine 2, Yangon (34/ERC-1,12-2017).

### 16S rRNA gene amplicon sequencing

DNA extraction from rectal swabs was performed as described previously (Additional File 1: Supplementary methods and materials) [18]. The V4 hypervariable region of the bacterial 16S rRNA gene was amplified as previously [19], and sequence read data was deposited in the EMBL European Nucleotide Archive (Accession Number: PRJEB39247). 16S rRNA sequence data were processed with QIIME2 (version 2018-2), using DADA2 inbuilt software for sequence modelling and taxonomic assignment was performed against the SILVA reference database (release 132) [20, 21]. Sequence data were subsampled to a uniform depth of 1900 reads, based on rarefaction curve asymptotes. Core microbiota were determined based on taxon prevalence (detection in more than 50% of individuals) at a median relative abundance of 0·01 or above.

### Shotgun metagenomic sequencing

Resistome composition was determined through shotgun metagenomic sequencing. Twenty-four patients were selected based on location and processed using Nextera XT DNA Library Prep Kit (Illumina Inc., CA, USA), and Nextera XT Index kit (Illumina Inc., CA, USA) in accordance with manufacturer instructions. An average of 13,977,146 ± 4,922,297 quality-filtered reads were obtained per sample. Sequence read data was deposited in the EMBL European Nucleotide Archive (Accession Number: PRJEB39247). Identification of antibiotic resistance genes was achieved through alignment-based homology searches against the Comprehensive Antibiotic Resistance Database (CARD), as previously described [22].

### Diversity measurements and statistical analysis

Two α-diversity indices were employed: taxon richness and Faith’s phylogenetic diversity using QIIME2 (version 2018-2). Between group comparisons were performed by permutational multivariate analysis of variance (PERMANOVA) and homogeneity of multivariate dispersions (PERMDISP) using PRIMER 7 (PRIMER-E Ltd, Plymouth, UK). Bray–Curtis similarity scores were determined from square root transformed relative abundances of taxa. Mann–Whitney U test was used for numerical comparisons (GraphPad Prism, version 8.2.1; GraphPad Software, La Jolla, California, USA) and the Spearman’s test was used for correlations (R software, version 3.5.1, Vienna, Austria).

### Distance-resistome similarity relationships

Inter-patient distance was scored using a categorical system (Additional File 2: Table S1). A resistome similarity matrix was generated using the Sørensen–Dice index and gene presence/absence data. DISTLM (DISTance based Linear Models; a distance-based regression analysis of univariate or multivariate data in response to predictor variables), using step-wise model selection for the *R*^*2*^ criterion, was used to analyse the relationship between inter-patient distance and the resistome (PRIMER 7, PRIMER-E Ltd, Plymouth, UK). Correction for multiple testing was performed using the Bonferroni method.

### Targeted AMR gene detection by quantitative PCR (qPCR)

Distribution of AMR genes that contributed to a distance-resistome similarity relationship (*NDM-7*, *OXA-1, dfrA14, catB3, fusB,* and *rmtB*), and three additional genes of high clinical relevance (*CTX-M-14, CMY-2, mcr-1.0*) was assessed by qPCR. Non-redundant representative sequences of each gene from the sequence data were aligned with reference sequences obtained from the CARD using the CLUSTAL OMEGA program [23], and to the NCBI database using the BLASTn module, to confirm specificity of gene annotation [24]. Gene carriage was determined by qPCR using SYBR Green assays (Additional File 3: Table S2). Sanger sequencing was used to confirm specificity of amplicon sequences that were more than 150 base pairs (*OXA-1, NDM-7, CTX-M-14, rmtB, dfrA14, fusB and mcr-1.0)*.

### Assessment of distance-resistome similarity relationships

Potential contributors to distance-resistome similarity relationships were investigated by univariate and multivariate regression using SAS statistical software (SAS University Edition, 2018). Multivariate analysis included common clinical variables that are associated with the development of antibiotic resistance genes; antibiotic use, conditions associated with broad spectrum antibiotic use (TB and sepsis), and length of hospital stay.

## Results

Our study was conducted in an open-plan adult general medicine ward with a total capacity of 95 beds (68 permanent and 27 temporary). The ward was divided into nine bays, of which one was a “private” bay, and one a high dependency unit. Thirteen temporary beds were situated in an adjacent corridor. There were no restrictions placed on patient movement within the ward, although patients varied in their mobility status. Patients within the ward shared the same toileting and showering facilities.

There were 60 resident inpatients (63% of total ward capacity, 88% of permanent ward capacity) the ward on the day of sample collection (Fig. 1a). One patient (temporary bed, Bay 1) could not be located and was excluded from the study. Patients were aged between 14 and 77 years and admitted for a range of indications (Table 1). Median length of stay was four days (IQR: 7·25 days). In the two weeks prior to sample collection, 43 patients (72·9%) had received either oral or intravenous antibiotics.

**Table 1:** Demographics and clinical parameters of subjects

Variable	Inpatients (n = 59)
Age-median (IQR)years	43 (27)
*Sex*	
Male	59 (100%)
*Recent antibiotic use (2 weeks)*	
Antibiotics	43 (72.9%)
No antibiotics	16 (27.1%)
*Route of antibiotics*	
Oral antibiotics	11 (25.6%)
Intravenous antibiotics	32 (74.4%)
Length of stay—median (IQR) days	4 (7.25)
*Reason for admission*	
Tuberculosis	16 (27.1%)
Liver disease (including alcohol related)	11 (18.6%)
Complications of diabetes mellitus	10 (16.9%)
Sepsis	7 (11.9%)
Hypertension	4 (6.8%)
Malignancy	3 (5.1%)
Ischaemic heart disease	3 (5.1%)
Others	5 (8.5%)
*Multiple co-morbidities*^a^	
Co-morbidity index 0–4	51(86.4%)
Co-morbidity index 5–8	8(13.6%)

^a^Charlson co-morbidity index used to assess impact of co-morbidities. Higher score indicates higher 10-year predictive mortality rate

### Rectal microbiota composition

All rectal samples were subjected to initial 16S rRNA gene amplicon sequencing (hereafter, 16S sequencing). One subject (permanent ward bed, Bay 1) was excluded from downstream analysis due to persistent low read depth. In total, 224 sequence variants were detected, representing 12 bacterial phyla. Of these, Firmicutes (51·5%), Actinobacteria (17·3%), Proteobacteria (14·2%), Bacteroidetes (11·1%) and Fusobacteria (1·3%) were predominant. Nine “core” bacterial genera were identified, of which seven were taxa associated with faeces (*Finegoldia*, *Anaerococcus*, *Enterococcus*, *Peptoniphilus*, *Escherichia*-*Shigella*, *Bacteroides*, *Streptococcus*) and two were taxa associated with the perianal region (*Staphylococcus*, *Corynebacterium*) (Additional File 12: Figure S1). No relationship was identified between the microbial composition of rectal swabs and the distance between patients (Spearman correlation r = − 0·02, *p* = 0·08, Additional File 13: Figure S2).

### Metagenomic characterisation of resistome features

Shotgun metagenomic analysis was performed on 24 representative patients (Fig. 1a). In total, 453 de novo assembled AMR genes were identified in rectal metagenomes. These included genes encoding multidrug efflux proteins (n = 173, 38.2%); and genes conferring resistance to glycopeptides (n = 52, 11.4%); beta-lactams (n = 37, 8.2%); macrolides (n = 33, 7.3%); aminoglycosides (n = 28, 6.2%); phenicol (n = 27, 6%); tetracyclines (n = 20, 4.4%); peptides (n = 17, 3.8%); diaminopyrimidine (n = 16, 3.5%) and other resistance traits (n = 50, 11%) (Additional File 4: Table S3).

In contrast to the resistance genes detected, the principal antibiotics prescribed during the study period were cephalosporins (23.7%) anti-TB medications (rifampicin, ethambutol, pyrazinamide, isoniazid) (20.3%), and fluoroquinolones (16.9%) (Additional File 5: Table S4).

### Association between bacterial taxa and AMR genes

The relative abundance of 453 resistance traits and 224 bacterial taxa, detected in the metagenomes of 24 subjects, were assessed by Spearman’s correlation. After adjustment for false discovery, 76 significant interactions were identified (*p* < 0.05, Additional File 6: Table S5). Apart from glycopeptide resistance genes, the majority of traits that were significantly correlated with bacterial taxa (beta-lactamase resistance, peptide resistance, or encoded efflux pumps) are commonly associated with Gram-negative bacteria. However, the taxa that were associated with these genes were predominantly Gram-positive anaerobic gut bacteria, belonging to the phylum Firmicutes. This observation may be the result of antibiotic use in this cohort. Cephalosporins (including combination cephalosporins) and fluoroquinolones represented 49.1% of total antibiotic use (Additional File 5: Table S4). These broad-spectrum are disproportionately active against Gram-negative bacteria, potentially resulting in a relative selection of Gram-positive anaerobes, and a concomitant selection of resistance determinants in Gram-negative taxa.

### Spatial resistome distribution

A resistome similarity matrix was generated based on the detection of resistance determinants within each patient (Additional File 14: Figure S3). Resistome similarity scores showed a weak but statistically significant positive correlation related with spatial distance similarity scores (r = 0.12, *p* = 0.04; Additional File 15: Figure S4). Exploratory DISTLM analysis identified 19 AMR genes that contributed significantly to the location-resistome relationship. These included genes conferring beta lactam resistance (n = 5), glycopeptide resistance (n = 3), efflux pump (n = 3), peptide antibiotics (n = 2), and others (n = 6) (Table 2).

**Table 2:** Resistance genes that contribute to spatial patterns within the sub-population where metagenomic analysis was performed

Gene	SS (trace)	Pseudo-F	*p* value	Explained variance (proportion)	Resistance mechanism
*OXA-1*	66.812	9.953	0.001	0.322	Beta-lactams resistance
*mcr-1.9*	56.881	7.916	0.004	0.274	Peptide antibiotic resistance
*BRP(MBL)*	55.229	7.603	0.005	0.266	Beta-lactams resistance
*dfrA14*	48.845	6.454	0.006	0.235	Diaminopyrimidine resistance
*omp38*	45.105	5.823	0.011	0.217	Beta-lactams resistance
*rmtB*	42.099	5.336	0.012	0.203	Aminoglycosides resistance
*NDM-7*	40.965	5.157	0.016	0.197	Beta-lactams resistance
*catB3*	40.085	5.020	0.014	0.193	Phenicol resistance
*vanSG*	37.290	4.593	0.024	0.180	Glycopeptide resistance
*tsnR*	36.330	4.450	0.017	0.175	Peptide antibiotic resistance
*mgtA*	36.330	4.450	0.015	0.175	Macrolide resistance
*mdfA*	36.019	4.404	0.023	0.173	Multidrug efflux pump
*kpnF*	32.752	3.930	0.037	0.158	Multidrug efflux pump
*vanN*	32.473	3.890	0.041	0.156	Glycopeptide resistance
*vanO*	31.383	3.736	0.048	0.151	Glycopeptide resistance
*CRP*	31.383	3.736	0.049	0.151	Multidrug efflux pump
*vgaE*	31.383	3.736	0.048	0.151	Streptogramin resistance
*fusB*	31.309	3.726	0.039	0.151	Fusidic acid resistance
*cfxA6*	30.898	3.668	0.046	0.149	Beta-lactams resistance

Performed using DISTLM test

*SS* sum of squares

The 19 AMR genes identified by DISTLM analysis were ranked by detection frequency. Those within the interquartile range (*OXA-1, NDM-7, dfrA14, catB3, fusB,* and *rmtB*) were further assessed by targeted qPCR analysis in the study population as a whole (n = 59) (Additional File 16: Figure S5). Three AMR genes which are of high clinical importance, but which did not show a location-distribution relationship (*CTX-M-14, CMY-2,* and *mcr-1.0*), were also included.

The results of targeted qPCR-based detection of *fusB* (40·7% of samples) *CMY-2* (31.0%), *NDM-7* (30.5%), *dfrA14* (28.8%), *CTX-M-14* (25.4%), *OXA-1* (24%), *catB3* (22%), *rmtB* (15.3%), and *mcr-1·0* (5.1%) (Additional File 7: Table S6) was consistent with resistome data, with metagenomic detection confirmed in > 96% of cases. DISTLM analysis based on qPCR data also confirmed the results of metagenomic analysis, with a significant relationship identified between patient location and AMR gene carriage for *OXA-1, NDM-7, dfrA14, catB3, fusB, and rmtB* genes. This relationship remained statistically significant after correction for false discovery for *catB3* (*p* = 0.002), *OXA-1* (*p* = 0.003), *NDM-7* (*p* = 0.047), and *dfrA14* (*p* = 0.0495) (Table 3).

**Table 3:** Resistance genes that contribute to spatial patterns within the study population

Gene	SS (trace)	Pseudo-F	*p* value	FDR adjusted (*p*)	Explained variance (proportion)
*catB3*	118.660	5.732	0.0002	0.002	0.091
*OXA-1*	106.230	5.078	0.0003	0.003	0.081
*NDM-7*	73.588	3.424	0.0052	0.047	0.057
*dfrA14*	76.405	3.564	0.0056	0.0495	0.059
*fusB*	60.368	2.779	0.0163	0.147	0.046
*rmtB*	52.082	2.382	0.0435	0.392	0.040
*CMY-2*	38.315	1.733	0.110	0.992	0.030
*mcr-1.0*	13.053	0.579	0.602	5.417	0.010
*CTX-M-14*	14.255	0.633	0.690	6.214	0.011

Performed using DISTLM test

*SS* sum of squares; *FDR* false discovery rate

### Prediction of distance-resistome relationships by clinical variables

**Fig. 2:**
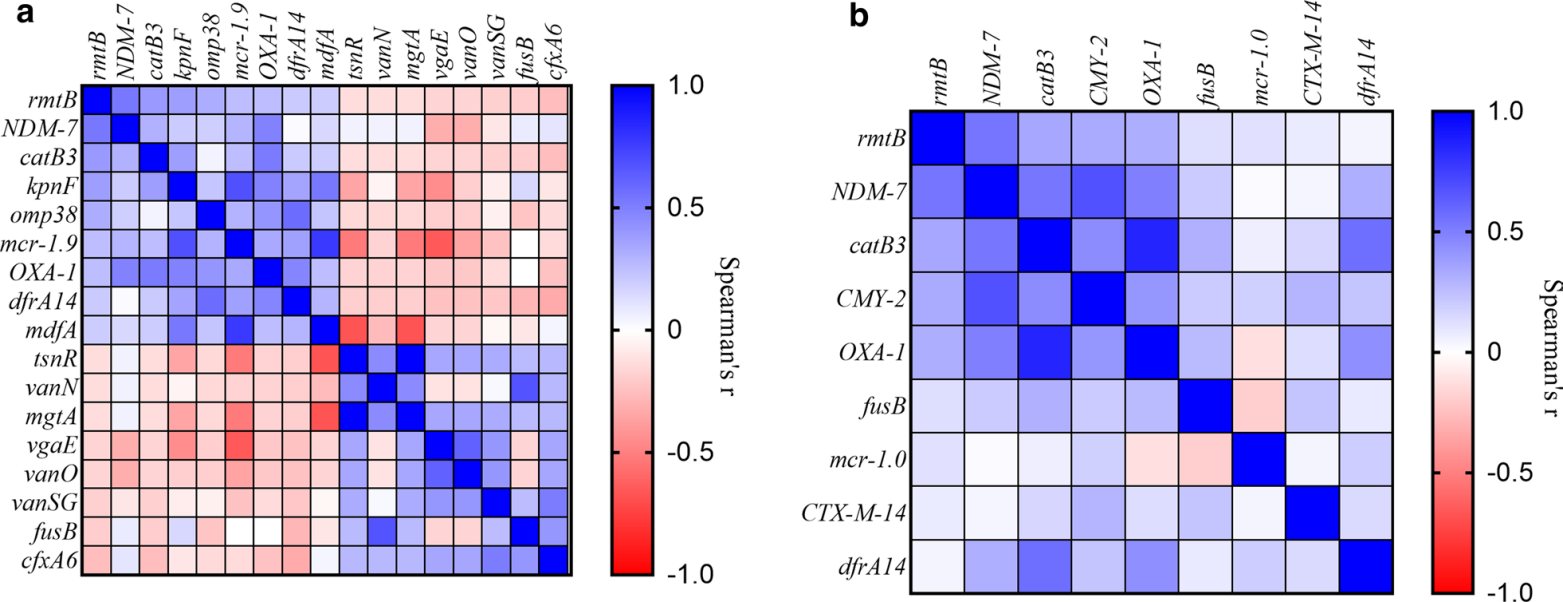
Heatmaps showing correlations between resistance genes that were identified as spatially associated from **a** shotgun metagenomic analysis of 24 individuals included in the sub-study and **b** quantitative PCR analysis of the entire study cohort. Performed using Spearman’s correlation on gene presence/absence

When AMR gene carriage was mapped against patient location, it was notable that the distribution of the genes identified as contributing to the distance-resistome relationships were disproportionately prevalent on one side of the ward (Bay 5–8) and in the corridor (Additional File 8: Table S7). Co-dispersion patterns of individual AMR genes were also identified, including a positive relationship between determinants that conferred resistance against antibiotics with Gram-negative activity (Fig. 2). While inter-patient transmission could explain such localised clustering, the deliberate co-location of patients with particular conditions (e.g. TB; Fig. 1B), and particular antibiotic exposures (e.g. antibiotics with Gram-negative activity) could result in such an effect. We therefore explored whether AMR dispersion might be explained by presence of sepsis, TB, MDR-TB, use of antibiotics or length of stay by using both univariate and multivariate approaches.

In age-adjusted univariate analysis, TB predicted the carriage of *OXA-1*(*p* = 0.02)*, catB3* (*p* =  < 0.01), and *dfrA14* (*p* < 0.01); length of stay in hospital predicted carriage of *NDM-7* (*p* = 0.02) and *CMY-2* (*p* = 0.04); and antibiotics use predicted carriage of *CMY-2* (*p* = 0.03) (Additional File 9: Table S8). TB still predicted the presence of *OXA-1* (*p* = 0.03)*, catB3* (*p* < 0·01)*, dfrA14* (*p* = 0.02), and *rmtB* (*p* = 0.03) in multivariate analysis, where age, sepsis and length of hospital stay were adjusted for*.* Length of stay predicted detection of *NDM-7* (*p* = 0.04) and *CMY-2* (*p* = 0.03) while bacterial sepsis predicted detection of *CMY-2* (*p* = 0.03) (Additional File 10: Table S9)*.*

We identified a number of resistance genes that were associated with patients with TB (*OXA-1, catB3, dfrA14* and *rmtB*). A likely contributor to this relationship was the longer average length of stay of TB patients compared to the wider patient population (median length of stay [IQR]: TB = 7.5 days [15.6]; non-TB = 4 days [6.0]), increasing the likelihood of AMR acquisition. We observed no differences in microbiota diversity or richness between TB and non-TB patients (Faith PD, *p* = 0.58; Observed OTUs, *p* = 0.36), or in overall community composition (PERMANOVA *p* = 0.30, PERMDISP *p* = 0.81). However, levels of Firmicutes were lower and levels of *Escherichia-Shigella* were significantly higher in those with TB (Additional File 17: Figure S6). *Escherichia-Shigella* was significantly correlated with *OXA-1, dfrA14* and *catB3* (Additional File 11: Table S10).

## Discussion

An improved understanding AMR transmission in South-East Asia is essential for the development of effective strategies to limit AMR spread. Selection pressures, reservoirs, and modes and routes of transmission vary considerably between settings. As a consequence, strategies developed in HICs may be inappropriate in LMIC. Despite this, LMIC responses to the threat of AMR spread are largely informed by studies in HIC settings. Local deployment of genomic technologies can provide valuable insight typically unavailable in low income regions.

Investigations of inter-patient AMR transmission usually focus on specific pathogens and resistance phenotypes. While such an approach can inform infection control strategies, it provides limited insight into the ongoing circulation of AMR determinants within patient populations. In contrast, metagenomic approaches provide several important advantages. First, they are able to identify AMR genes inclusively, without the need for a priori target selection. Second, they are able to assess relationships between resistance carriage and patient variables at a resistome level, reducing the impact of individual determinants or bacterial species. Finally, objective identification of individual resistance determinants enables targeted analysis by qPCR. Indeed, metagenomics-based approaches have also been employed successfully in a number of other contexts, including to map opportunistic pathogens and AMR genes in a tertiary hospital environment in Singapore [25], and to determine the non-nosocomial circulation of AMR determinants in low-income communities [26].

In our study population, resistome analysis revealed a diverse range of AMR determinants. A substantial number of these genes contributed to a significant location-resistome relationship, in which their co-carriage in separate individuals varied inversely with inter-patient distance. Such a relationship is consistent with patient-to-patient AMR transmission, assuming that transmission risk increases with increasing patient proximity. Even after correction for false discovery, significant spatial distribution patterns existed for clinically important AMR genes, including *OXA-1* and *NDM-7*.

Importantly, transmission was not the primary driver of patient location-resistome relationship.

When we explored the distribution of AMR genes associated with significant spatial distribution, we found that TB predicted carriage of genes that showed significant relationships with inter-patient distance, including *OXA-1, catB3,* and *dfrA14*. The contribution of other variables such as antibiotic therapy, sepsis and length of stay were explored and demonstrated that length of stay predicted *NDM-7* and *CMY-2*, and bacterial sepsis predicted *CMY-2*.

Closer examination revealed that placement of TB patients was largely limited to one quarter of the ward. Our findings, therefore, suggest that the observed resistome-location relationships are likely to result largely from patient placement. While our assessment did not demonstrate a wider transmission event within the ward, it is possible that a higher frequency of environmental and direct inter-patient transmission of resistance genes may have contributed to the observed patterns of AMR gene carriage within patients with TB.

Patient length of stay in hospital was closely associated with *NDM-7* and *CMY-2*. These genes both confer broad-spectrum β-lactam resistance typically associated with prolonged antibiotic exposure. The relationship may therefore be due to cumulative exposure to antibiotics provided in hospital. Similarly, an observed association between *CMY-2* and sepsis, in addition to the relationship with length of stay, could reflect prolonged high antibiotic exposure, or the clinical impact of sepsis causes by pathogens that carry *NDM-7* or *CMY-2*.

The relationship between TB and carriage of *OXA-1, catB3, rmtB* and *dfrA14* is likely to reflect the impact of gut microbiota disruption arising as a result of prolonged hospital stay and antibiotic therapy. A previous study has reported the relative abundance of gut Firmicutes to be decreased in those with TB, and the prevalence of Proteobacteria to be increased [27]. We observed a reduction in Firmicutes and an increase in *Escherichia-Shigella*, with the latter correlated with *OXA-1, dfrA14* and *catB3.*

Notably, our analysis identified a number of AMR genes that are of considerable clinical concern, including *OXA-1* and *NDM-7*. Carbapenem-resistant organisms are a major health threat, particularly in LMICs [28], and *NDM* and *OXA* genes have emerged as key contributors to carbapenem resistance in multidrug resistant Gram-negative pathogens [29]. The fact that patients are likely to have brought bacteria carrying these AMR determinants into the hospital environment within their intestinal microbiota highlights the importance of gaining a better understanding of AMR carriage and transmission within the wider Myanmar population.

Consideration of AMR carriage tends to focus on point-source infection outbreaks in hospitals or within the wider community. However, the resistome associated with the intestinal microbiome is constantly being contributed to and reshaped by antibiotic and non-antibiotic exposures. The acquisition of an AMR-carrying bacterial strain or resistant determinant might precede overt infection by a substantial period, particularly where the determinant is carried within a commensal species prior to migrating into a pathogen population. Such dynamics could explain differences between the types of AMR gene detected in our study population and contemporary antibiotic exposure of patients. Other than multidrug efflux pumps, AMR determinants identified in our study most commonly conferred resistance to glycopeptides, followed by beta-lactams, macrolides, and aminoglycosides. In contrast, the principal antibiotic exposures of patients at the time of the study were cephalosporins, anti-TB medications, and fluoroquinolones. Such a disparity has been reported previously in other resource-limited settings [29] and highlights the role of AMR transmission beyond the hospital setting in defining the patient intestinal resistome.

Prior to metagenomic analysis, we also applied 16S sequencing to rectal swab DNA, both to confirm sample quality and to enable relation of resistome traits to microbial diversity. AMR determinants can move between bacterial populations through horizontal gene transfer. As a result, microbiota and resistome traits are not always strongly aligned, and indeed, intestinal microbiota depletion can result in increased acquisition of resistant bacteria from the environment [30]. However, in our study population, microbiota diversity was not significantly correlated with the number of AMR genes carried and was unrelated to patient location.

Our study had limitations that should be considered. We chose not to assess AMR reservoirs such as fomites within the hospital environment, or potential mediators of transmission, such as clinical staff and inter-patient interaction. While potentially informative, it was decided to instead focus first on determining whether evidence of substantial transmission existed. We did not assess whether common resistance determinants in co-located patients were identical, a process that cannot be readily achieved through the metagenomic approach employed. As the focus of our study was AMR gene detection as a marker of a potentially transmissible trait, we did not assess phenotypic resistance conferred by AMR markers, or which bacterial species carried them. Finally, our study was cross-sectional in nature and involved a single ward within an individual hospital. As such, further longitudinal analysis, performed across multiple settings, would provide additional insight into potential causality in associations between AMR gene distribution and patient variables.

Despite these limitations, our use of a metagenomic strategy to define AMR carriage within hospital inpatients in a resource-limited setting did not identify substantial inter-patient transmission. These findings are consistent with effective infection control, which is critical in reducing risks of nosocomial outbreaks. However, they do highlight the need for wider assessments of AMR carriage and transmission beyond the hospital environment as a basis for establishing evidence-based national AMR prevention and containment strategies. The development of effective strategies to reduce AMR transmission at a population level would likely provide substantial benefit, not least by reducing AMR carriage in patients being admitted to hospital which can then be transmitted within the nosocomial environment. Metagenomic analysis represents a powerful means to generate the data needed to inform such measures.

## Supplementary Information

**Additional file 1:** Supplementary methods and materials

**Additional file 2: Table S1.** Patient co-location categories that were assigned to determine spatial relation between patients.

**Additional file 3: Table S2.** Primer sequences of antibiotic resistance and positive control (16S rRNA) genes for quantitative PCR.

**Additional file 4: Table S3.** Antimicrobial resistance genes identified through shotgun metagenomics.

**Additional file 5: Table S4.** Antibiotic use according to class of antibiotic during study period and annual hospital use 2017-2018.

**Additional file 6: Table S5.** Significant correlations between detected AMR genes and bacterial taxa (n=24 patients)

**Additional file 7: Table S6.** Proportion of genes detected in shotgun metagenomic sequencing and qPCR analysis.

**Additional file 8: Table S7.** Distribution of resistance genes according to patient location on the ward.

**Additional file 9: Table S8.** Univariate analysis of clinical variables and resistance genes.

**Additional file 10: Table S9.** Multivariate regression analysis of clinical variables and resistance genes.

**Additional file 11: Table S10.** Correlation between relative abundance of specific taxa and absolute abundance of associated AMR genes.

**Additional file 12: Figure S1.** Identification of core microbiota among study population (>50% population, median>0.01).

**Additional file 13: Figure S2.** Spearman’s correlation of spatial distance between individuals located on the ward (spatial distance) and compositional similarity distance between individual’s microbiota (weighted UNIFRAC distance).

**Additional file 14: Figure S3.** Distribution of detected resistance genes within patient cohort. The number of patients (of a total of 24) in whom a specific number of resistance were detected is shown. Genes that detected in all patients, or in only a single individual only, were excluded from analysis of potential inter-patient transmission.

**Additional file 15: Figure S4.** Spearman’s correlation of spatial distance between individuals located on the ward (spatial distance) and resistance gene presence/absence similarity (resistance distance). (A) Resistance genes present in patients located within the bay and corridor (B) resistance genes present in patients located within the bay.

**Additional file 16: Figure S5.** (A) Overall distribution of resistance genes identified through DISTLM according to their prevalence (B) Prevalence of individual genes as detected by metagenomic analysis. Genes present in >15% and <50% were selected for validation by quantitative PCR.

**Additional file 17: Figure S6.** (A) Levels of Firmicutes between patients with and without TB (B) Levels of *Escherichia-Shigella* between patients with and without TB.

## Data Availability

The sequence read data was deposited in the EMBL European Nucleotide Archive (Accession Number: PRJEB39247).

## Abbreviations

AMR: Antimicrobial resistance
LMIC: Low- and middle-income countries
WHO: World Health Organization
GDP: Gross domestic product
DALY: Disability-adjusted life year
HIC: High-income countries
SAHMRI: South Australian Health and Medical Research Institute
CARD: Comprehensive Antibiotic Resistance Database
DISTLM: DISTance based Linear Models
TB: Tuberculosis
MDR-TB: Multi-drug resistant TB
