# Supplementary material for: Investigating potential transmission of antimicrobial resistance in an open-plan hospital ward: a cross-sectional metagenomic study of resistome dispersion in a lower middle-income setting
Source: Antimicrob Resist Infect Control. 2021 Mar 18;10:56. doi: 10.1186/s13756-021-00915-w (PMC7977308; doi:10.1186/s13756-021-00915-w)
Supplement: Supplementary file 1 — Additional file 1: Supplementary methods and materials [file 13756_2021_915_MOESM1_ESM.docx]

**Supplementary methods**

**Swab processing**

Rectal swabs were immediately stored frozen at -80°C until DNA extraction. To maximise yield, 400 µL of sterile (10mM Tris, 1mM EDTA) Tris-EDTA (TE) buffer (ROCHE, Victoria, Australia) was added to the swabs and vortexed for 30 sec and solution recovered from swabs by centrifugation (3374 x g for 5 minutes). Swabs were then discarded, and DNA extraction performed on TE solution.

**DNA extraction**

TE solution containing sample was heated to 95 °C for 1 minute, before being cooled at 4°C for 2 minutes. Lysozyme (ROCHE, ThermoFisher Scientific, Victoria, Australia) and lysostaphin (Sigma-Aldrich, MO, USA) were then added to a final concentration of 2·9 mg/mL and 0.1 mg/mL, respectively, and samples incubated at 37 °C for 1 hr. Proteinase K (Fermentas, ThermoFisher Scientific, Victoria, Australia) and sodium dodecyl sulphate (Sigma-Aldrich, MO, USA) were then added to a final concentration of 1·2 mg/mL and 1·5 %, w/v, respectively.

Following incubation for 1 hr at 50 °C, approximately 0·13 g of washed beads (1:1 of 0·1mm and 1mm silica zirconium beads) was added to the sample prior to a cycle of bead beating at 6·5 m/s for 1 min using a FastPrep-24 bead beater (MP Biomedicals, Santa Ana, USA). The supernatant was transferred into a new 2 mL conical screw cap microtube and 100 µl of 5M sodium chloride and 600 µl of phenol:chloroform:isoamyl alcohol (25:24:1; saline buffered at pH8·0; Sigma-Aldrich, MO, USA) were added and samples vortexed for 60 sec. The aqueous-organic layers were separated by centrifugation at 13,000 x g for 20 minutes and 600 µl of the aqueous layer was transferred to a new 2 mL microfuge tube. DNA was precipitated by the addition of 10 M ammonium acetate and 100% ethanol (Sigma Aldrich, MO, USA) in a 1:10 and 1:1 ratio with sample volume, respectively and recovered using an EZ-10 Spin column in accordance with manufacturer’s instructions (Bio Basic, Inc., Ontario, Canada). DNA was eluted in 50 µl UltraPure DNase/RNase-free distilled water (Gibco, ThermoFisher Scientific, Victoria, Australia) three times (total 150 µl) and samples were vacuum concentrated until pellet formation using CHRIST-RVC 2-18 CDplus machine (John Morris Group, Germany). DNA pellets were resuspended in 100 µL UltraPure DNase/RNase-free distilled water. Samples were then stored at -80 °C prior to analysis.

**16S rRNA gene amplicon sequencing**

The V4 hypervariable region of the bacterial 16S rRNA gene was amplified from OP swab DNA using modified universal bacterial primer pairs 515F (5'-TCGTCGGCAGCGTCAGATGTGTATAAGAGACAGGTGCCAGCMGCCGCGGTAA-3') and 806R (5'- GTCTCGTGGGCTCGGAGATGTGTATAAGAGACAGGGACTACHVGGGTWTCTAA-3'). with Illumina adapter overhang sequences as indicated by underline. Amplicons were generated, cleaned, indexed and sequenced according to the Illumina MiSeq 16S Metagenomic Sequencing Library Preparation protocol(<https://support.illumina.com/documents/documentation/chemistry_documentation/16s/16s-metagenomic-library-prep-guide-15044223-b.pdf>) with certain modifications. Briefly, an initial PCR reaction contained at least 12·5 ng of DNA, 0·5 μL of forward primer (1 μM), 0·5 μL of reverse primer (1 μM) and 12·5 μL of 2× KAPA HiFi Hotstart ReadyMix (KAPA Biosystems, Wilmington, MA, USA) in a total volume of 25 μL. The PCR reaction was performed on a Veriti 96-well Thermal Cycler (Life Technologies) using the following program: 95 °C for 3 min, followed by 25 cycles of 95 °C for 30 sec, 55 °C for 30 sec and 72 °C for 30 sec and a final extension step at 72 °C for 5 min. Samples were multiplexed using a dual-index approach with the Nextera XT Index kit (Illumina Inc., San Diego, CA, USA) according to the manufacturer’s instructions. The final library was paired end sequenced at 2 × 300 bp using a MiSeq Reagent Kit v3 on an Illumina MiSeq platform. Sequencing was performed at the David R Gunn Genomics Facility, South Australian Health and Medical Research Institute.

**Sequence data processing**

The Quantitative Insights Into Microbial Ecology (QIIME2-2018·2) software was used to analyse the 16S rRNA sequence generated from paired-end sequence reads using the QIIME2 bioinformatics pipeline (<https://docs.qiime2.org/2018.11/tutorials/moving-pictures>). Single-nucleotide variants (SNVs) were assigned to the reads against the SILVA database release 132 (December 2017).

**Metagenomic bioinformatic processing**

Illumina paired-end reads were adapter- and quality-filtered using Trimmomatic v0·38 [1]. These high-quality interleaved reads were used for *de novo* assembly of contigs of at least 900 bp with IDBA-UD v1.1.1 [2]. Gene prediction was performed using MetaGeneMark [3], with genes shorter than 100 bp discarded. A non-redundant gene catalogue of 1,538,641 genes was constructed using CD-HIT [4] with parameters: “-c 0·95 –aS 0·9” (genes with greater than 95% identity and aligned length covering over 90% of the shorter gene were grouped together). Genes with greater than 100 bp were retained and transcribed into amino acids using transeq, part of The European Molecular Biology Open Software Suite (EMBOSS v6.5.7) [5]. The transcribed non-redundant gene catalogue was annotated to the comprehensive antibiotic resistance database (CARD) release 2020-03-04 [6], using BLASTP, with parameters: “-task blastp-fast -evalue 1e-10 -qcov_hsp_perc 99 -max_hsps 1 -max_target_seqs 1.” High quality reads from each sample were aligned against the gene catalogue, and gene-length normalized read counts were calculated using SOAP [7]. For every sample, relative gene abundances were estimated by dividing the number of the gene-length normalized read counts for each gene by the total of reads that uniquely mapped to any gene in the catalogue as previously described [8].

**Quantitation of total bacterial load, resistance gene carriage and specific bacterial taxa**

Levels of *OXA-1, NDM-7, fusB, rmtB, dfrA14, catB3, mcr-1, CTX-M-14* and *CMY-2* genes were assessed using SYBR Green assays based on previously described primer pairsand previous described conditions [9]. Modifications to the qPCR annealing conditions were used for *mcr-1, NDM-7* and *OXA-1* qPCR assays (Table S2). For SYBR Green qPCR assays, 1 μL of DNA extract, 0.2 μM of each primer, 17.5 μL of 2X Platinum SYBR Green qPCR SuperMix-UDG (Invitrogen, Carlsbad, USA) and the appropriate volume of water was added to a 35 μL total reaction volume. Quantitative RT-PCR were performed on three technical replicates, at 10 μL reaction volume per replicate, on a QuantStudio 6 and 7 Flex Real-Time PCR system (Applied Biosystems, Carlsbad, USA). Cycling conditions for SYBR Green qPCR assays were: 50°C for 2 min, 95°C for 10 min, followed by 40 cycles of 95°C for 15 secs and 60°C for 1 min. Melt curve analysis was then performed at the following conditions: 95°C for 15 secs, followed by an initial stage temperature of 60°C for 1 min and a final temperature of 95°C for 15 secs, with readings recorded at increments of 0·05°C/s. Standard curves were generated for each qPCR reaction based on serial dilutions of DNA from bacterial isolates previously identified using whole genome sequencing.

**References**

1. Bolger AM, Lohse M, Usadel B. Trimmomatic: a flexible trimmer for Illumina sequence data. Bioinformatics 2014; 30(15): 2114-20.

2. Peng Y, Leung HC, Yiu SM, Chin FY. IDBA-UD: a de novo assembler for single-cell and metagenomic sequencing data with highly uneven depth. Bioinformatics 2012; 28(11): 1420-8.

3. Zhu W, Lomsadze A, Borodovsky M. Ab initio gene identification in metagenomic sequences. Nucleic Acids Res 2010; 38(12): e132-e.

4. Li W, Godzik A. Cd-hit: a fast program for clustering and comparing large sets of protein or nucleotide sequences. Bioinformatics 2006; 22(13): 1658-9.

5. Rice P, Longden I, Bleasby A. EMBOSS: the European Molecular Biology Open Software Suite. Trends Genet 2000; 16(6): 276-7.

6. Alcock BP, Raphenya AR, Lau TTY, et al. CARD 2020: antibiotic resistome surveillance with the comprehensive antibiotic resistance database. Nucleic Acids Res 2019; 48(D1): D517-D25.

7. Li R, Yu C, Li Y, et al. SOAP2: an improved ultrafast tool for short read alignment. Bioinformatics 2009; 25(15): 1966-7.

8. Taylor SL, Leong LEX, Mobegi FM, et al. Long-Term Azithromycin Reduces *Haemophilus influenzae* and Increases Antibiotic Resistance in Severe Asthma. Am J Respir Crit Care Med 2019; 200(3): 309-17.

9. Choo JM, Abell GCJ, Thomson R, et al. Impact of Long-Term Erythromycin Therapy on the Oropharyngeal Microbiome and Resistance Gene Reservoir in Non-Cystic Fibrosis Bronchiectasis. mSphere 2018; 3(2): e00103-18.

10. Wang F-H, Qiao M, Su J-Q, Chen Z, Zhou X, Zhu Y-G. High Throughput Profiling of Antibiotic Resistance Genes in Urban Park Soils with Reclaimed Water Irrigation. Environ Sci Technol

2014; 48(16): 9079-85.

11. Bert F, Branger C, Lambert-Zechovsky N. Identification of PSE and OXA beta-lactamase genes in *Pseudomonas aeruginosa* using PCR-restriction fragment length polymorphism. J Antimicrob Chemother 2002; 50(1): 11-8.

12. Xu J, He F. Characterization of a *NDM-7* carbapenemase-producing *Escherichia coli* ST410 clinical strain isolated from a urinary tract infection in China. Infect Drug Resist 2019; 12: 1555-64.

13. Dallenne C, Da Costa A, Decré D, Favier C, Arlet G. Development of a set of multiplex PCR assays for the detection of genes encoding important β-lactamases in *Enterobacteriaceae*. J Antimicrob Chemother 2010; 65(3): 490-5.

14. Rebelo AR MH, Cavaco L, Bortolaia V, Kjeldgaard JS, Hendriksen RS. Multiplex PCR for detection of plasmid-mediated colistin resistance determinants, *mcr-1, mcr-2, mcr-3, mcr-4* and *mcr-5* for surveillance purposes. Euro Surveill 2018; 23(6): 17-00672.

15. Bossé JT, Li Y, Walker S, et al. Identification of *dfrA14* in two distinct plasmids conferring trimethoprim resistance in *Actinobacillus pleuropneumoniae.* J Antmicrob Chemother 2015; 70(8): 2217-22.

16. Doi Y, Arakawa Y. 16S Ribosomal RNA Methylation: Emerging Resistance Mechanism against Aminoglycosides. Clin Infect Dis 2007; 45(1): 88-94.

17. Liu Y, Geng W, Yang Y, et al. Susceptibility to and resistance determinants of fusidic acid in *Staphylococcus aureus* isolated from Chinese children with skin and soft tissue infections. FEMS Immunol Med Microbiol 2012; 64(2): 212-8.

18. Roberts MC. Update on acquired tetracycline resistance genes. FEMS Microbiol Lett 2005; 245(2): 195-203.

19. Wang Y, Taylor DE. Chloramphenicol resistance in *Campylobacter coli*: nucleotide sequence, expression, and cloning vector construction. Gene 1990; 94(1): 23-8.

20. LeBlanc DJ, Lee LN, Titmas BM, Smith CJ, Tenover FC. Nucleotide sequence analysis of tetracycline resistance gene *tetO* from *Streptococcus mutans* DL5. J Bacteriol 1988; 170(8): 3618-26.

21. Brolund A, Sundqvist M, Kahlmeter G, Grape M. Molecular Characterisation of Trimethoprim Resistance in *Escherichia coli* and *Klebsiella pneumoniae* during a Two Year Intervention on Trimethoprim Use. PloS one 2010; 5(2): e9233.

22. Schwarz S, Spies U, Cardoso M. Cloning and sequence analysis of a plasmid-encoded chloramphenicol acetyltransferase gene from *Staphylococcus intermedius.* J Gen Microbiol 1991; 137(4): 977-81.

23. Speer BS, Bedzyk L, Salyers AA. Evidence that a novel tetracycline resistance gene found on two *Bacteroides* transposons encodes an NADP-requiring oxidoreductase. J Bacteriol 1991; 173(1): 176-83.

24. Cocconcelli PS, Cattivelli D, Gazzola S. Gene transfer of vancomycin and tetracycline resistances among *Enterococcus faecalis* during cheese and sausage fermentations. Int J Food Microbiol 2003; 88(2): 315-23.

25. Charpentier E, Courvalin P. Emergence of the trimethoprim resistance gene *dfrD* in *Listeria monocytogenes* BM4293. Antimicrob Agents Chemother 1997; 41(5): 1134-6.

26. Allignet J, el Solh N. Diversity among the gram-positive acetyltransferases inactivating streptogramin A and structurally related compounds and characterization of a new staphylococcal determinant, *vatB*. Antimicrob Agents Chemother 1995; 39(9): 2027-36.

27. Garnier F, Taourit S, Glaser P, Courvalin P, Galimand M. Characterization of transposon Tn1549, conferring *VanB*-type resistance in *Enterococcus* spp.The GenBank accession number for the 33803 bp sequence of Tn1549 is AJ192329. Microbiology 2000; 146(6): 1481-9.

28. Zhao J, Aoki T. Cloning and nucleotide sequence analysis of a chloramphenicol acetyltransferase gene from *Vibrio anguillarum*. Microbiol Immunol 1992; 36(7): 695-705.

29. Scaria J, Warnick LD, Kaneene JB, May K, Teng C-H, Chang Y-F. Comparison of phenotypic and genotypic antimicrobial profiles in *Escherichia coli* and *Salmonella enterica* from the same dairy cattle farms. Mol Cell Probes 2010; 24(6): 325-45.

30. Fraimow H, Knob C, Herrero IA, Patel R. Putative *VanRS*-like two-component regulatory system associated with the inducible glycopeptide resistance cluster of *Paenibacillus popilliae*. Antimicrob Agents Chemother 2005; 49(7): 2625-33.

31. Lyras D, Rood JI. Transposition of Tn4451 and Tn4453 involves a circular intermediate that forms a promoter for the large resolvase, TnpX. Mol Microbiol 2000; 38(3): 588-601.

32. Ambrose KD, Nisbet R, Stephens DS. Macrolide efflux in *Streptococcus pneumoniae* is mediated by a dual efflux pump (mel and mef) and is erythromycin inducible. Antimicrob Agents Chemother 2005; 49(10): 4203-9.

33. Kim HB, Wang M, Park CH, Kim EC, Jacoby GA, Hooper DC. oqxAB encoding a multidrug efflux pump in human clinical isolates of *Enterobacteriaceae*. Antimicrob Agents Chemother 2009; 53(8): 3582-4.

34. Partridge SR, Kwong SM, Firth N, Jensen SO. Mobile Genetic Elements Associated with Antimicrobial Resistance. Clin Microbiol Rev 2018; 31(4): e00088-17.

35. Liu J, Keelan P, Bennett PM, Enne VI. Characterization of a novel macrolide efflux gene, *mef(B)*, found linked to sul3 in porcine *Escherichia coli.* J Antimicrob Chemother 2009; 63(3): 423-6.

36. Xu X, Lin D, Yan G, et al. *vanM*, a new glycopeptide resistance gene cluster found in *Enterococcus faecium*. Antimicrob Agents Chemother 2010; 54(11): 4643-7.

37. Jung Y-H, Shin ES, Kim O, et al. Characterization of Two Newly Identified Genes, *vgaD* and *vatG*, Conferring Resistance to Streptogramin A in *Enterococcus faecium*. Antimicrob Agents Chemother 2010; ;54(11):4744-9.

38. Lebreton F, Depardieu F, Bourdon N, et al. D-Ala-d-Ser *VanN*-type transferable vancomycin resistance in *Enterococcus faecium*. Antimicrob Agents Chemother 2011; 55(10): 4606-12.

39. Ito T, Katayama Y, Hiramatsu K. Cloning and Nucleotide Sequence Determination of the Entire mec DNA of Pre-Methicillin-Resistant *Staphylococcus aureus* N315. Antimicrob Agents Chemother 1999; 43(6): 1449.

40. Trieu-Cuot P, Courvalin P. Nucleotide sequence of the *Streptococcus faecalis* plasmid gene encoding the 3'5"-aminoglycoside phosphotransferase type III. Gene 1983; 23(3): 331-41.

41. Hansen LH, Planellas MH, Long KS, Vester B. The order *Bacillales* hosts functional homologs of the worrisome *cfr* antibiotic resistance gene. Antimicrob Agents Chemother 2012; 56(7): 3563-7.

42. Pinto-Alphandary H, Mabilat C, Courvalin P. Emergence of aminoglycoside resistance genes *aadA* and *aadE* in the genus *Campylobacter*. Antimicrob Agents Chemother 1990; 34(6): 1294-6.

43. Bertsch D, Uruty A, Anderegg J, Lacroix C, Perreten V, Meile L. Tn6198, a novel transposon containing the trimethoprim resistance gene *dfrG* embedded into a Tn916 element in *Listeria monocytogenes*. J Antimicrob Chemother 2013; 68(5): 986-91.

44. Harrison EM, Paterson GK, Holden MTG, et al. A novel hybrid SCCmec-mecC region in *Staphylococcus sciuri*. J Antimicrob Chemother 2014; 69(4): 911-8.

45. Nonaka L, Maruyama F, Miyamoto M, Miyakoshi M, Kurokawa K, Masuda M. Novel conjugative transferable multiple drug resistance plasmid pAQU1 from *Photobacterium damselae* subsp. damselae isolated from marine aquaculture environment. Microbes Environ 2012; 27(3): 263-72.

46. Scott KP, Melville CM, Barbosa TM, Flint HJ. Occurrence of the New Tetracycline Resistance Gene *tet (W)* in Bacteria from the Human Gut. Antimicrob Agents Chemother 2000; 44(3): 775.

47. Schwarz FV, Perreten V, Teuber M. Sequence of the 50-kb conjugative multiresistance plasmid pRE25 from *Enterococcus faecalis* RE25. Plasmid 2001; 46(3): 170-87.

48. Leng Z, Riley DE, Berger RE, Krieger JN, Roberts MC. Distribution and mobility of the tetracycline resistance determinant *tetQ*. J Antimicrob Chemother 1997; 40(4): 551-9.

49. Kehrenberg C, Ojo KK, Schwarz S. Nucleotide sequence and organization of the multiresistance plasmid pSCFS1 from *Staphylococcus sciuri*. J Antimicrob Chemother 2004; 54(5): 936-9.

50. Kehrenberg C, Schwarz S. *dfrA20,* A novel trimethoprim resistance gene from *Pasteurella multocida*. Antimicrob Agents Chemother 2005; 49(1): 414-7.

51. Akhtar M, Hirt H, Zurek L. Horizontal transfer of the tetracycline resistance gene *tetM* mediated by pCF10 among *Enterococcus faecalis* in the house fly (Musca domestica L.) alimentary canal. Microb Ecol 2009; 58(3): 509-18.

52. Abril C, Brodard I, Perreten V. Two novel antibiotic resistance genes, *tet(44)* and *ant(6)-Ib*, are located within a transferable pathogenicity island in *Campylobacter fetus subsp. fetu*s. Antimicrob Agents Chemother 2010; 54(7): 3052-5.

53. Kadlec K, Schwarz S. Identification of a novel trimethoprim resistance gene, *dfrK*, in a methicillin-resistant *Staphylococcus aureus* ST398 strain and its physical linkage to the tetracycline resistance gene *tet(L).* Antimicrob Agents Chemother 2009; 53(2): 776-8.

54. Schwendener S, Perreten V. New transposon Tn6133 in methicillin-resistant *Staphylococcus aureus* ST398 contains *vga(E)*, a novel streptogramin A, pleuromutilin, and lincosamide resistance gene. Antimicrob Agents Chemother 2011; 55(10): 4900-4.

55. Daigle DM, Hughes DW, Wright GD. Prodigious substrate specificity of *AAC(6')-APH(2")*, an aminoglycoside antibiotic resistance determinant in enterococci and staphylococci. Chem Biol 1999; 6(2): 99-110.

56. Paterson GK, Harrison EM, Holmes MA. The emergence of mecC methicillin-resistant *Staphylococcus aureus*. Trends Microbiol 2014; 22(1): 42-7.

57. Shore AC, Lazaris A, Kinnevey PM, et al. First Report of cfr-Carrying Plasmids in the Pandemic Sequence Type 22 Methicillin-Resistant *Staphylococcus aureus* Staphylococcal Cassette Chromosome mec Type IV Clone. Antimicrob Agents Chemother 2016; 60(5): 3007.

58. He T, Wang R, Liu D, et al. Emergence of plasmid-mediated high-level tigecycline resistance genes in animals and humans. Nat Microbiol 2019; 4(9): 1450-6.

59. Martin P, Jullien E, Courvalin P. Nucleotide sequence of *Acinetobacter baumannii aphA-6* gene: evolutionary and functional implications of sequence homologies with nucleotide-binding proteins, kinases and other aminoglycoside-modifying enzymes. Mol Microbiol 1988; 2(5): 615-25.

60. Bauernfeind A, Stemplinger I, Jungwirth R, Giamarellou H. Characterization of the plasmidic beta-lactamase *CMY-2,* which is responsible for cephamycin resistance. Antimicrob Agents Chemother 1996; 40(1): 221-4.

61. Rands CM, Starikova EV, Brüssow H, Kriventseva EV, Govorun VM, Zdobnov EM. ACI-1 beta-lactamase is widespread across human gut microbiomes in *Negativicutes* due to transposons harboured by tailed prophages. Environ Microbiol 2018; 20(6): 2288-300.

62. Morin CJ, Patel PC, Levesque RC, Letarte R. Monoclonal antibodies to *TEM-1* plasmid-mediated beta-lactamase. Antimicrob Agents Chemother 1987; 31(11): 1761-7.

63. Kehrenberg C, Schwarz S. fexA, a novel *Staphylococcus lentus* gene encoding resistance to florfenicol and chloramphenicol. Antimicrob Agents Chemother 2004; 48(2): 615-8.

64. Fernández-Martínez M, Ruiz Del Castillo B, Lecea-Cuello MJ, Rodríguez-Baño J, Pascual Á, Martínez-Martínez L. Prevalence of Aminoglycoside-Modifying Enzymes in *Escherichia coli* and *Klebsiella pneumoniae* Producing Extended Spectrum β-Lactamases Collected in Two Multicenter Studies in Spain. Microb Drug Resist 2018; 24(4): 367-76.

65. Mobaraki S, Aghazadeh M, Soroush Barhaghi MH, et al. Prevalence of integrons 1, 2, 3 associated with antibiotic resistance in *Pseudomonas aeruginosa* isolates from Northwest of Iran. Biomedicine (Taipei) 2018; 8(1): 2.

66. Usui M, Kajino A, Kon M, et al. Prevalence of 16S rRNA methylases in Gram-negative bacteria derived from companion animals and livestock in Japan. J Vet Med Sci 2019; 81(6): 874-8.

67. Bouallègue-Godet O, Ben Salem Y, Fabre L, et al. Nosocomial outbreak caused by *Salmonella enterica* serotype Livingstone producing CTX-M-27 extended-spectrum beta-lactamase in a neonatal unit in Sousse, Tunisia. J Clin Microbiol 2005; 43(3): 1037-44.

68. Peirano G, Agersø Y, Aarestrup FM, dos Reis EMF, dos Prazeres Rodrigues D. Occurrence of integrons and antimicrobial resistance genes among *Salmonella enterica* from Brazil. J Antimicrob Chemother 2006; 58(2): 305-9.

69. Kadlec K, Pomba CF, Couto N, Schwarz S. Small plasmids carrying *vga(A)* or *vga(C)* genes mediate resistance to lincosamides, pleuromutilins and streptogramin A antibiotics in methicillin-resistant *Staphylococcus aureus* ST398 from swine. J Antimicrob Chemother 2010; 65(12): 2692-3.

70. Laraki N, Galleni M, Thamm I, et al. Structure of In31, a blaIMP-containing *Pseudomonas aeruginosa* integron phyletically related to In5, which carries an unusual array of gene cassettes. Antimicrob Agents Chemother 1999; 43(4): 890-901.

71. Mazodier P, Cossart P, Giraud E, Gasser F. Completion of the nucleotide sequence of the central region of Tn5 confirms the presence of three resistance genes. Nucleic Acids Res 1985; 13(1): 195-205.

72. Zhang G, Leclercq SO, Tian J, et al. A new subclass of intrinsic aminoglycoside nucleotidyltransferases, *ANT(3")-II*, is horizontally transferred among *Acinetobacter spp.* by homologous recombination. PLoS Genet 2017; 13(2): e1006602.

73. Khan SA, Nawaz MS, Khan AA, Cerniglia CE. Transfer of Erythromycin Resistance from Poultry to Human Clinical Strains of *Staphylococcus aureus.* J Clin Microbiol 2000; 38(5): 1832.

74. Doi Y, Wachino J-I, Yamane K, et al. Spread of novel aminoglycoside resistance gene *aac(6')-Iad* among *Acinetobacter* clinical isolates in Japan. Antimicrob Agents Chemother 2004; 48(6): 2075-80.

75. Heim U, Tietze E, Weschke W, Tschäpe H, Wobus U Nucleotide sequence of a plasmid born streptothricin-acetyl-transferase gene *(sat-1)*. Nucleic Acids Res 1989; 17(17): 7103.

76. Ito R, Mustapha MM, Tomich AD, et al. Widespread Fosfomycin Resistance in Gram-Negative Bacteria Attributable to the Chromosomal *fosA* Gene. mBio 2017; 8(4): e00749-17.

77. Razavi M, Marathe NP, Gillings MR, Flach CF, Kristiansson E, Joakim Larsson DG. Discovery of the fourth mobile sulfonamide resistance gene. Microbiome 2017; 5(1): 160.

78. Manageiro V, Clemente L, Romão R, et al. IncX4 Plasmid Carrying the New mcr-1.9 Gene Variant in a *CTX-M-8*-Producing *Escherichia coli* Isolate Recovered From Swine. Front Microbiol 2019; 10:367.

79. Wang X, Wang Y, Zhou Y, et al. Emergence of a novel mobile colistin resistance gene, *mcr-8,* in NDM-producing *Klebsiella pneumoniae*. Emerg Microbes Infect 2018; 7(1): 122.

80. Ceccarelli D, Bani S, Cappuccinelli P, Colombo MM. Prevalence of *aadA1* and *dfrA15* class 1 integron cassettes and SXT circulation in *Vibrio cholerae* O1 isolates from Africa. J Antimicrob Chemother 2006; 58(5): 1095-7.

81. Partridge SR, Tsafnat G, Coiera E, Iredell JR. Gene cassettes and cassette arrays in mobile resistance integrons. FEMS Microbiol Rev 2009; 33(4): 757-84.

82. Wang Y, Lv Y, Cai J, et al. A novel gene, *optrA,* that confers transferable resistance to oxazolidinones and phenicols and its presence in *Enterococcus faecalis* and Enterococcus faecium of human and animal origin. J Antimicrob Chemother 2015; 70(8): 2182-90.

83. Liu YY, Wang Y, Walsh TR, et al. Emergence of plasmid-mediated colistin resistance mechanism *MCR-1* in animals and human beings in China: a microbiological and molecular biological study. Lancet Infect Dis 2016; 16(2): 161-8.

84. Ramirez MS, Nikolaidis N, Tolmasky M. Rise and dissemination of aminoglycoside resistance: the *aac(6′)-Ib* paradigm. Front Microbiol 2013; 4:121.

85. Fu Z, Liu Y, Chen C, et al. Characterization of Fosfomycin Resistance Gene, fosB, in Methicillin-Resistant *Staphylococcus aureus* Isolates. PloS one 2016; 11(5): e0154829.

86. Leclercq SO, Wang C, Zhu Y, et al. Diversity of the Tetracycline Mobilome within a Chinese Pig Manure Sample. Appl Environ Microbiol 2016; 82(21): 6454.

87. Shrestha S, Tada T, Shrestha B, et al. Emergence of Aminoglycoside Resistance Due to armA methylase in Multi-drug Resistant *Acinetobacter Baumannii* Isolates in a University Hospital in Nepal. J Nepal Health Res Counc 2016; 14(33): 72-6.

88. Thungapathra M, Amita, Sinha KK, et al. Occurrence of antibiotic resistance gene cassettes *aac(6')-Ib, dfrA5, dfrA12,* and *ereA2* in class I integrons in non-O1, non-O139 *Vibrio cholerae* strains in India. Antimicrob Agents Chemother 2002; 46(9): 2948-55.

89. Liu Y, Feng Y, Wu W, et al. First Report of *OXA-181*-Producing *Escherichia Coli* in China and Characterization of the Isolate Using Whole-Genome Sequencing. Antimicrob Agents Chemother 2015; 59(8): 5022.

90. Zangenah S, Andersson AF, Özenci V, Bergman P. Genomic analysis reveals the presence of a class D beta-lactamase with broad substrate specificity in animal bite associated *Capnocytophaga* species. Eur J Clin Microbiol Infect Dis 2017; 36(4): 657–662.

91. Sugumar M, Kumar KM, Manoharan A, Anbarasu A, Ramaiah S. Detection of *OXA-1* β-Lactamase Gene of *Klebsiella pneumoniae* from Blood Stream Infections (BSI) by Conventional PCR and In-Silico Analysis to Understand the Mechanism of OXA Mediated Resistance. PloS one 2014; 9(3): e91800.

92. Yan H, Yu R, Li D, et al. A novel multiresistance gene cluster located on a plasmid-borne transposon in *Listeria monocytogenes.* J Antimicrob Chemother 2020; 75(4): 868-72.

93. Göttig S, Hamprecht AG, Christ S, Kempf VAJ, Wichelhaus TA. Detection of *NDM-7* in Germany, a new variant of the New Delhi metallo-β-lactamase with increased carbapenemase activity. J Antimicrob Chemother 2013; 68(8): 1737-40.

94. Tosini F, Visca P, Luzzi I, et al. Class 1 integron-borne multiple-antibiotic resistance carried by IncFI and IncL/M plasmids in *Salmonella enterica* serotype typhimurium. Antimicrob Agents Chemother 1998; 42(12): 3053-8.

95. Petinaki E, Guérin-Faublée V, Pichereau V, et al. Lincomycin resistance gene *lnu(D)* in *Streptococcus uberis*. Antimicrob Agents Chemother 2008; 52(2): 626-30.

96. Wang Y, Lo W-U, Lai EL, Chow K-H, Ho P-L. Complete Sequence of the Multidrug-Resistant IncL/M Plasmid pIMP-HB623 Cocarrying Bla IMP-34 and fosC2 in an *Enterobacter Cloacae* Strain Associated With Medical Travel to China. Antimicrob Agents Chemother 2015; 59(9): 5854.

97. Kumburu HH, Sonda T, van Zwetselaar M, et al. Using WGS to identify antibiotic resistance genes and predict antimicrobial resistance phenotypes in MDR *Acinetobacter baumannii* in Tanzania. J Antimicrob Chemother 2019; 74(6): 1484-93.

98. Márquez C, Labbate M, Ingold AJ, et al. Recovery of a functional class 2 integron from an *Escherichia coli* strain mediating a urinary tract infection. Antimicrob Agents Chemother 2008; 52(11): 4153-4.

99. Perreten V, Boerlin P. A new sulfonamide resistance gene (*sul3*) in *Escherichia coli* is widespread in the pig population of Switzerland. Antimicrob Agents Chemother 2003; 47(3): 1169-72.

100. Hata M, Suzuki M, Matsumoto M, et al. Cloning of a novel gene for quinolone resistance from a transferable plasmid in *Shigella flexner*i 2b. Antimicrob Agents Chemother 2005; 49(2): 801-3.

101. Levings RS, Hall RM, Lightfoot D, Djordjevic SP. *linG,* a new integron-associated gene cassette encoding a lincosamide nucleotidyltransferase. Antimicrob Agents Chemother 2006; 50(10): 3514-5.

102. Sandalli C, Buruk CK, Sancaktar M, Ozgumus OB. Prevalence of integrons and a new *dfrA17* variant in Gram-negative bacilli which cause community-acquired infections. Microbiol Immunol 2010; 54(3): 164-9.

103. Novotna G, Janata J. A new evolutionary variant of the streptogramin A resistance protein, Vga(A)LC, from *Staphylococcus haemolyticus* with shifted substrate specificity towards lincosamides. Antimicrob Agents Chemother 2006; 50(12): 4070-6.

104. Blackwell GA, Hall RM. The tet39 Determinant and the *msrE-mphE* Genes in *Acinetobacter* Plasmids Are Each Part of Discrete Modules Flanked by Inversely Oriented pdif (XerC-XerD) Sites. Antimicrob Agents Chemother 2017; 61(8): e00780-17.

105. Kiratisin P, Apisarnthanarak A, Saifon P, Laesripa C, Kitphati R, Mundy LM. The emergence of a novel ceftazidime-resistant CTX-M extended-spectrum beta-lactamase, *CTX-M-55,* in both community-onset and hospital-acquired infections in Thailand. Diagn Microbiol Infect Dis 2007; 58(3): 349-55.

106. Hormeño L, Ugarte-Ruiz M, Palomo G, et al. *ant(6)-I* Genes Encoding Aminoglycoside O-Nucleotidyltransferases Are Widely Spread Among Streptomycin Resistant Strains of *Campylobacter jejuni* and *Campylobacter coli.* Front Microbiol 2018; 9:2515.

107. Hua X, Pan C, Zhu L, et al. Complete genome sequence of *Acinetobacter baumannii* A1296 (ST1469) with a small plasmid harbouring the *tet(39)* tetracycline resistance gene. *J* Glob Antimicrob Resist 2017; 11: 105-7.

108. Brown S, Amyes SG. The sequences of seven class D beta-lactamases isolated from carbapenem-resistant *Acinetobacter baumannii* from four continents. Clin Microbiol Infect 2005; 11(4): 326-9

109. Ceccarelli D, Salvia AM, Sami J, Cappuccinelli P, Colombo MM. New cluster of plasmid-located class 1 integrons in *Vibrio cholerae* O1 and a *dfrA15* cassette-containing integron in *Vibrio parahaemolyticus* isolated in Angola. Antimicrob Agents Chemother 2006; 50(7): 2493-9.

110. Jacoby GA, Walsh KE, Mills DM, et al. *qnrB*, another plasmid-mediated gene for quinolone resistance. Antimicrob Agents Chemother 2006; 50(4): 1178-82.

111. Pai H, Byeon J-h, Yu S, Lee BK, Kim S. *Salmonella enterica* serovar typhi strains isolated in Korea containing a multidrug resistance class 1 integron. Antimicrob Agents Chemother 2003; 47(6): 2006-8.

112. Sáenz Y, Briñas L, Domínguez E, et al. Mechanisms of resistance in multiple-antibiotic-resistant *Escherichia coli* strains of human, animal, and food origins. Antimicrob Agents Chemother 2004; 48(10): 3996-4001.

113. Jemima SA, Verghese S. *SHV-28*, an extended-spectrum beta-lactamase produced by a clinical isolate of *Klebsiella pneumoniae* in south India. Indian J Med Microbiol 2009; 27(1): 51-4.

114. Cloeckaert A, Baucheron S, Flaujac G, et al. Plasmid-mediated florfenicol resistance encoded by the *floR* gene in *Escherichia coli* isolated from cattle. Antimicrob Agents Chemother 2000; 44(10): 2858-60.

115. Sköld O. Resistance to trimethoprim and sulfonamides. Vet Res 2001; 32(3-4): 261-73.

116. Sandvang D. Novel streptomycin and spectinomycin resistance gene as a gene cassette within a class 1 integron isolated from *Escherichia coli*. Antimicrob Agents Chemother 1999; 43(12): 3036-8.

117. Chanawong A, M'Zali FH, Heritage J, Xiong J-H, Hawkey PM. Three cefotaximases, *CTX-M-9, CTX-M-13*, and *CTX-M-14,* among *Enterobacteriaceae* in the People's Republic of China. Antimicrob Agents Chemother 2002; 46(3): 630-7.

118. Tauch A, Zheng Z, Pühler A, Kalinowski J. *Corynebacterium striatum* chloramphenicol resistance transposon Tn5564: genetic organization and transposition in *Corynebacterium glutamicum*. Plasmid 1998; 40(2): 126-39.

119. Jacob J, Evers S, Bischoff K, Carlier C, Courvalin P. Characterization of the sat4 gene encoding a streptothricin acetyltransferase in *Campylobacter coli* BE/G4. FEMS Microbiol Lett 1994; 120(1-2): 13-7.

120. Allignet J, El Solh N. Characterization of a new staphylococcal gene, *vgaB*, encoding a putative ABC transporter conferring resistance to streptogramin A and related compounds. Gene 1997; 202(1-2): 133-8.

121. Daly M, Villa L, Pezzella C, Fanning S, Carattoli A. Comparison of multidrug resistance gene regions between two geographically unrelated *Salmonella* serotypes. J Antimicrob Chemother 2005; 55(4): 558-61.

122. Cameron FH, Groot Obbink DJ, Ackerman VP, Hall RM. Nucleotide sequence of the *AAD(2'')* aminoglycoside adenylyltransferase determinant aadB. Evolutionary relationship of this region with those surrounding *aadA* in R538-1 and dhfrII in R388. Nucleic Acids Res 1986; 14(21): 8625-35.

123. Tribuddharat C, Fennewald M. Integron-mediated rifampin resistance in *Pseudomonas aeruginosa.* Antimicrob Agents Chemother 1999; 43(4): 960-2.

124. Charpentier E, Gerbaud G, Courvalin P. Presence of the *Listeria t*etracycline resistance gene tet(S) in *Enterococcus faecalis.* Antimicrob Agents Chemother 1994; 38(10): 2330-5.

125. Lüthje P, von Köckritz-Blickwede M, Schwarz S. Identification and characterization of nine novel types of small staphylococcal plasmids carrying the lincosamide nucleotidyltransferase gene lnu(A). J Antimicrob Chemother 2007; 59(4): 600-6.

126. Tannock GW, Luchansky JB, Miller L, et al. Molecular characterization of a plasmid-borne (pGT633) erythromycin resistance determinant (ermGT) from *Lactobacillus reuteri* 100-63. Plasmid 1994; 31(1): 60-71.

127. Tenover FC, Filpula D, Phillips KL, Plorde JJ. Cloning and sequencing of a gene encoding an aminoglycoside 6'-N-acetyltransferase from an R factor of *Citrobacter diversus.* *J* Bacteriol 1988; 170(1): 471-3.
