# Supplementary material for: Investigating potential transmission of antimicrobial resistance in an open-plan hospital ward: a cross-sectional metagenomic study of resistome dispersion in a lower middle-income setting
Source: Antimicrob Resist Infect Control. 2021 Mar 18;10:56. doi: 10.1186/s13756-021-00915-w (PMC7977308; doi:10.1186/s13756-021-00915-w)
Supplement: Supplementary file 2 — Additional file 2: Table S1. Patient co-location categories that were assigned to determine spatial relation between patients. [file 13756_2021_915_MOESM2_ESM.docx]

**Table S1:** Patient co-location categories that were assigned to determine spatial relation between patients.

| **Patient location** | **Category no.** |
| --- | --- |
| Patient located within the same bay | 1 |
| Patient located in the neighbouring bay | 2 |
| Patient located in the opposite bay from the index bay | 3 |
| Patient located in the diagonally opposite bay from the index bay | 4 |
| Patient located two bays away from the index bay | 5 |
| Patient located in a diagonal bay, two bays away from index bay | 6 |
| Patient located three bays away from the index bay | 7 |
| Patient located in a diagonal bay, three bays away from index bay | 8 |
| Patient located furthest away from the index bay | 9 |
| Patient located furthest away diagonally from the index bay | 10 |
| Patient located in the corridor in comparison to patient located in a bay | 11 |
