# Supplementary material for: Investigating potential transmission of antimicrobial resistance in an open-plan hospital ward: a cross-sectional metagenomic study of resistome dispersion in a lower middle-income setting
Source: Antimicrob Resist Infect Control. 2021 Mar 18;10:56. doi: 10.1186/s13756-021-00915-w (PMC7977308; doi:10.1186/s13756-021-00915-w)
Supplement: Supplementary file 3 — Additional file 3: Table S2. Primer sequences of antibiotic resistance and positive control (16S rRNA) genes for quantitative PCR. [file 13756_2021_915_MOESM3_ESM.docx]

**Table S2:** Primer sequences of antibiotic resistance and positive control (16S rRNA) genes for quantitative PCR.

| **Gene** | **Sequence (5' - 3')** | **Annealing conditions (temperature, extension time)** | **References** |
| --- | --- | --- | --- |
| *OXA-1* | CGGATGGTTTGAAGGGTTTATTAT  TTTCTTGGCTTTTATGCTTG | 55°C, 60 s | [10, 11] |
| *NDM-7* | GAATGTCTGGCAGCACACTT  GCATTGGCATAAGTCGCAATCC | 56°C, 60 s | [12]  This study |
| *CTX-M-14* | TCAAGCCTGCCGATCTGGT  TGATTCTCGCCGCTGAAG | 60°C, 60 s | [13] |
| *CMY-2* | AAAGCCTCATGGGTGCATAAA  ATAGCTTTTGTTTGCCAGCATCA | 60°C, 60 s | [10] |
| *mcr-1.0* | AGTCCGTTTGTTCTTGTGGC  AGATCCTTGGTCTCGGCTTG | 56°C, 60 s | [14] |
| *dfrA14* | GCTGCGAAAGCGAAAAACGGCGT  ATCGTCGATAAGTGGAGCGTAGA  GGC | 60°C, 60 s | This study  [15] |
| *catB3* | GCACTCGATGCCTTCCAAAA  AGAGCCGATCCAAACGTCAT | 60°C, 60 s | [10] |
| *rmtB* | GCTTTCTGCGGGCGATGTAA  ATGCAATGCCGCGCTCGTAT | 60°C, 60 s | [16] |
| *fusB* | TGAACTACCTAGTCCGCAA  TTATATATTTCCGATTTGATGCAAG | 60°C, 60 s | This study  [17] |
