# Supplementary material for: Investigating potential transmission of antimicrobial resistance in an open-plan hospital ward: a cross-sectional metagenomic study of resistome dispersion in a lower middle-income setting
Source: Antimicrob Resist Infect Control. 2021 Mar 18;10:56. doi: 10.1186/s13756-021-00915-w (PMC7977308; doi:10.1186/s13756-021-00915-w)
Supplement: Supplementary file 4 — Additional file 4: Table S3. Antimicrobial resistance genes identified through shotgun metagenomics. [file 13756_2021_915_MOESM4_ESM.docx]

**Table S3**: Antimicrobial resistance genes identified through shotgun metagenomics

| **Gene name** | **Gene accession ID** | **ARO number** | **ARO class** | **Evidence of gene mobility** | **Reference for**  **gene mobility** |
| --- | --- | --- | --- | --- | --- |
| *tetB(P)* | AAA20117.1 | ARO:3000195 | Tetracycline resistance | Yes | [18] |
| *cat type A-13* | AAA23018.1 | ARO:3004454 | Phenicol resistance | Yes | [19] |
| *tetO* | AAA23033.2 | ARO:3000190 | Tetracycline resistance | Yes | [20] |
| *dfrA3* | AAA25550.1 | ARO:3003105 | Diaminopyrimidine resistance | Yes | [21] |
| *cat type A-7* | AAA26615.1 | ARO:3004457 | Phenicol resistance | Yes | [22] |
| *tetX* | AAA27471.1 | ARO:3000205 | Tetracycline resistance | Yes | [23] |
| *vanRA* | AAA65953.1 | ARO:3002919 | Glycopeptide resistance | Yes | [24] |
| *vanSA* | AAA65954.1 | ARO:3002931 | Glycopeptide resistance | Yes | [24] |
| *vanHA* | AAA65955.1 | ARO:3002942 | Glycopeptide resistance | Yes | [24] |
| *vanA* | AAA65956.1 | ARO:3000010 | Glycopeptide resistance | Yes | [24] |
| *dfrD* | AAA85213.1 | ARO:3002866 | Diaminopyrimidine resistance | Yes | [25] |
| *vatB* | AAA86871.1 | ARO:3002841 | Streptogramin resistance | Yes | [26] |
| *vanRB* | AAB05622.1 | ARO:3002921 | Glycopeptide resistance | Yes | [27] |
| *vanSB* | AAB05623.1 | ARO:3002932 | Glycopeptide resistance | Yes | [27] |
| *vanYB* | AAB05624.1 | ARO:3002956 | Glycopeptide resistance | Yes | [27] |
| *vanWB* | AAB05625.1 | ARO:3002964 | Glycopeptide resistance | Yes | [27] |
| *vanHB* | AAB05626.1 | ARO:3002943 | Glycopeptide resistance | Yes | [27] |
| *cat type A-14* | AAB23649.1 | ARO:3004460 | Phenicol resistance | Yes | [28] |
| *carA* | AAC32027.1 | ARO:3002817 | Macrolide resistance | Yes | [29] |
| *vanHF* | AAF36802.1 | ARO:3002945 | Glycopeptide resistance | Yes | [30] |
| *vanF* | AAF36803.1 | ARO:3002908 | Glycopeptide resistance | Yes | [30] |
| *catD* | AAF66228.1 | ARO:3002682 | Phenicol resistance | Yes | [31] |
| *vatE* | AAF86220.1 | ARO:3002844 | Streptogramin resistance | Yes | [26] |
| *mefE* | AAK99775.1 | ARO:3000614 | Macrolide resistance | Yes | [32] |
| *oqxB* | AAP43110.2 | ARO:3003923 | Fluoroquinolone resistance | Yes | [33] |

| *vanSF* | AAR84673.1 | ARO:3002936 | Glycopeptide resistance | Yes | [30] |
| --- | --- | --- | --- | --- | --- |
| *blaR1* | ABU39979.1 | ARO:3000217 | Beta-lactam resistance | Yes | [34] |
| *mefB* | ACJ63262.1 | ARO:3003107 | Macrolide resistance | Yes | [35] |
| *vanRM* | ACL82957.1 | ARO:3002928 | Glycopeptide resistance | Yes | [36] |
| *vanSM* | ACL82958.1 | ARO:3002939 | Glycopeptide resistance | Yes | [36] |
| *vanHM* | ACL82960.1 | ARO:3002947 | Glycopeptide resistance | Yes | [36] |
| *vanM* | ACL82961.1 | ARO:3002911 | Glycopeptide resistance | Yes | [36] |
| *vgaD* | ACX92986.2 | ARO:3002832 | Streptogramin resistance | Yes | [37] |
| *vanN* | AEP40500.1 | ARO:3002912 | Glycopeptide resistance | Yes | [38] |
| *vanTN* | AEP40502.2 | ARO:3002975 | Glycopeptide resistance | Yes | [38] |
| *vanRN* | AEP40503.1 | ARO:3002929 | Glycopeptide resistance | Yes | [38] |
| *vanSN* | AEP40504.1 | ARO:3002940 | Glycopeptide resistance | Yes | [38] |
| *mecA* | AGC51118.1 | ARO:3000617 | Beta-lactam resistance | Yes | [39] |
| *APH(3')-IIIa* | AGV10830.1 | ARO:3002647 | Aminoglycoside resistance | Yes | [40] |
| *clbA* | AGZ55247.1 | ARO:3002814 | Macrolide-lincosamide-linezolid-phenicol-streptogramin resistance | Yes | [41] |
| *ANT(6)-Ia* | AHE40557.1 | ARO:3002626 | Aminoglycoside resistance | Yes | [42] |
| *clbC* | BAD63613.1 | ARO:3002816 | Linezolid resistance | Yes | [41] |
| *dfrG* | BAE15963.1 | ARO:3002868 | Diaminopyrimidine resistance | Yes | [43] |
| *mecB* | BAI83385.1 | ARO:3003440 | Beta-lactam resistance | Yes | [44] |
| *mefC* | BAL43360.1 | ARO:3003745 | Macrolide resistance | Yes | [45] |
| *tetW* | CAA10975.1 | ARO:3000194 | Tetracycline resistance | Yes | [46] |
| *cat type A-7* | CAA63498.2 | ARO:3004458 | Phenicol resistance | Yes | [47] |
| *tetQ* | CAA79727.1 | ARO:3000191 | Tetracycline resistance | Yes | [48] |
| *lsaB* | CAE18141.1 | ARO:3003111 | Clindamycin resistance | Yes | [49] |
| *dfrA20* | CAE53424.1 | ARO:3003016 | Diaminopyrimidine resistance | Yes | [50] |
| *tetM* | CAQ49384.1 | ARO:3000186 | Tetracycline resistance | Yes | [51] |
| *tet44* | CBH51823.1 | ARO:3000556 | Tetracycline resistance | Yes | [52] |
| *ANT(6)-Ib* | CBH51824.1 | ARO:3002629 | Aminoglycoside resistance | Yes | [52] |
| *dfrK* | CBL80435.1 | ARO:3002869 | Diaminopyrimidine resistance | Yes | [53] |
| *vgaE* | CBY88983.1 | ARO:3002833 | Streptogramin resistance | Yes | [54] |
| *tetA* | NP_862226.1 | ARO:3004639 | Tetracycline resistance | Yes | [18] |
| *AAC(6')-Ie-APH(2'')-Ia* | AAA88548.1 | ARO:3002597 | Aminoglycoside resistance | Yes | [55] |
| *mecC* | CCC86795.1 | ARO:3001209 | Beta-lactam resistance | Yes | [56] |
| *cfr(B)* | CDF47262.1 | ARO:3004649 | Macrolide-lincosamide-streptogramin resistance | Yes | [57] |
| *tet(X4)* | QBQ69719.1 | ARO:3004720 | Tetracycline resistance | Yes | [58] |
| *APH(3')-VIa* | CAA30578.1 | ARO:3002652 | Aminoglycoside resistance | Yes | [59] |
| *CMY-2* | CAA62957.1 | ARO:3002013 | Beta-lactam resistance | Yes | [60] |
| *ACI-1* | CAB51471.1 | ARO:3004359 | Beta-lactam resistance | Yes | [61] |
| *TEM-1* | CAD09800.1 | ARO:3000873 | Beta-lactam resistance | Yes | [62] |
| *fexA* | CAD70268.1 | ARO:3002704 | Efflux pump conferring antibiotic resistance | Yes | [63] |
| *APH(3')-Ia* | CAE51638.1 | ARO:3002641 | Aminoglycoside resistance | Yes | [64] |
| *catB10* | CAI47810.1 | ARO:3003110 | Phenicol resistance | Yes | [65] |
| *rmtB* | CAP07796.1 | ARO:3000860 | Aminoglycoside resistance | Yes | [66] |
| *dfrA21* | CAP69659.1 | ARO:3003017 | Diaminopyrimidine resistance | Yes | [67] |
| *dfrA22* | CAX16467.1 | ARO:3003018 | Diaminopyrimidine resistance | Yes | [68] |
| *vgaC* | CBL58195.1 | ARO:3002831 | Streptogramin resistance | Yes | [69] |
| *clbB* | BAH45481.1 | ARO:3002815 | Macrolide-streptogramin-lincosamide resistance | Yes | [41] |
| *catB6* | CAA11473.1 | ARO:3002678 | Phenicol resistance | Yes | [70] |
| *APH(6)-Ic* | CAA25854.1 | ARO:3002659 | Aminoglycoside resistance | Yes | [71] |
| *ANT(3'')-IIa* | CAA26199.1 | ARO:3004089 | Aminoglycoside resistance | Yes | [72] |
| *ermA* | CAA26964.1 | ARO:3000347 | Macrolide-lincosamide-streptogramin resistance | Yes | [73] |
| *AAC(6')-Iad* | BAD12078.1 | ARO:3002572 | Aminoglycoside resistance | Yes | [74] |
| *sat1* | BAD95494.1 | ARO:3002895 | Nucleoside antibiotic resistance | Yes | [75] |
| *fosA6* | AMQ12811.1 | ARO:3004111 | Fosfomycin resistance | Yes | [76] |
| *sul4* | AUI09862.1 | ARO:3004361 | Sulfonamide antibiotic resistance | Yes | [77] |
| *mcr-1.9* | AVA31022.1 | ARO:3004507 | Peptide antibiotic resistance | Yes | [78] |
| *mcr-8* | AVX52225.1 | ARO:3004516 | Peptide antibiotic resistance | Yes | [79] |
| *dfrA15* | AHB39758.1 | ARO:3003013 | Diaminopyrimidine resistance | Yes | [80] |
| *catB11* | AID93387.1 | ARO:3004660 | Phenicol resistance | Yes | [81] |
| *optrA* | AKA86814 | ARO:3003746 | Mactrolide-streptogramin-lincosamide | Yes | [82] |
| *mcr-1* | AKF16168 | ARO:3003689 | Peptide antibiotic resistance | Yes | [83] |
| *AAC(6')-Ib7* | AKN19287.1 | ARO:3002578 | Aminoglycoside resistance | Yes | [84] |
| *fosB4* | ALM24139.1 | ARO:3004671 | Fosfomycin resistance | Yes | [85] |
| *tet(W/N/W)* | AMP42147.1 | ARO:3004442 | Tetracycline resistance | Yes | [86] |
| *armA* | ADC55560.1 | ARO:3000858 | Aminoglycoside resistance | Yes | [87] |
| *dfrA12* | ADG84870.1 | ARO:3002858 | Diaminopyrimidine resistance | Yes | [88] |
| *OXA-181* | AEP16366.1 | ARO:3001784 | Beta-lactam resistance | Yes | [89] |
| *OXA-347* | AET35493.1 | ARO:3001777 | Beta-lactam resistance | Yes | [90] |
| *OXA-1* | AFB82783.1 | ARO:3001396 | Beta-lactam resistance | Yes | [91] |
| *catA8* | AFN69318.1 | ARO:3004658 | Phenicol resistance | Yes | [92] |
| *NDM-7* | AFQ31613.1 | ARO:3002357 | Beta-lactam resistance | Yes | [93] |
| *catB3* | AFQ93498.1 | ARO:3002676 | Phenicol resistance | Yes | [94] |
| *lnuD* | ABR14060.1 | ARO:3002838 | Lincosamide resistance | Yes | [95] |
| *AAC(3)-IId* | ABS70977.1 | ARO:3004623 | Aminoglycoside resistance | Yes | [96] |
| *msrE* | ACB05808.1 | ARO:3003109 | Macrolide,streptogramin,lincosamide resistance | Yes | [97] |
| *dfrA14* | ACI32877.1 | ARO:3002859 | Diaminopyrimidine resistance | Yes | [98] |
| *sul3* | ACJ63260.1 | ARO:3000413 | Sulfonamide resistance | Yes | [99] |
| *qnrS1* | ABF47469.1 | ARO:3002790 | Fluoroquinolone resistance | Yes | [100] |
| *linG* | ABG65740.1 | ARO:3002879 | Lincosamide resistance | Yes | [101] |
| *dfrA17* | ABG91835.1 | ARO:3002860 | Diaminopyrimidine resistance | Yes | [102] |
| *vgaALC* | ABH10964.1 | ARO:3002830 | Streptogramin resistance | Yes | [103] |
| *mphE* | ABI20451.1 | ARO:3003741 | Macrolide resistance | Yes | [104] |
| *CTX-M-55* | ABI34705.1 | ARO:3001917 | Beta-lactam resistance | Yes | [105] |
| *aad(6)* | AAU10334.1 | ARO:3002628 | Aminoglycoside resistance | Yes | [106] |
| *tet(39)* | AAW66497.1 | ARO:3000566 | Efflux pump conferring antibiotic resistance | Yes | [107] |
| *OXA-68* | AAW81339.1 | ARO:3001616 | Beta-lactam resistance | Yes | [108] |
| *qacH* | AAZ42322.1 | ARO:3003836 | Efflux pump conferring antibiotic resistance | Yes | [109] |
| *dfrA5* | ABB89122.1 | ARO:3002861 | Diaminopyrimidine resistance | Yes | [88] |
| *qnrB1* | ABC86904.2 | ARO:3002714 | Fluroquinolone resistance | Yes | [110] |
| *mel* | AAL73129.1 | ARO:3000616 | Efflux pump conferring antibiotic resistance | Yes | [32] |
| *tet(D)* | AAL75563.1 | ARO:3000168 | Efflux pump conferring antibiotic resistance | Yes | [18] |
| *tet(A)* | AAN06707.1 | ARO:3000165 | Efflux pump conferring antibiotic resistance | Yes | [18] |
| *catB8* | AAO52851.1 | ARO:3004456 | Phenicol resistance | Yes | [111] |
| *dfrA1* | AAP74961.2 | ARO:3002854 | Diaminopyrimidine resistance | Yes | [112] |
| *SHV-28* | AAG15384.1 | ARO:3001086 | Beta-lactam resistance | Yes | [113] |
| *floR* | AAG16656.1 | ARO:3002705 | Phenicol resistance | Yes | [114] |
| *sul2* | AAL59753.1 | ARO:3000412 | Sulfonamide resistance | Yes | [115] |
| *tet(J)* | AAD12753.1 | ARO:3000177 | Efflux pump conferring antibiotic resistance | Yes | [18] |
| *aadA5* | AAF17880.1 | ARO:3002605 | Aminoglycoside resistance | Yes | [116] |
| *CTX-M-14* | AAF72530.1 | ARO:3001877 | Beta-lactam resistance | Yes | [117] |
| *cmx* | AAG03380.1 | ARO:3002703 | Efflux pump conferring antibiotic resistance | Yes | [118] |
| *tet(K)* | AAB28795.1 | ARO:3000178 | Efflux pump conferring antibiotic resistance | Yes | [18] |
| *sat4* | AAB53445.1 | ARO:3002897 | Nucleoside antibiotic | Yes | [119] |
| *vgaB* | AAB95639.1 | ARO:3000118 | Streptogramin resistance | Yes | [120] |
| *aadA3* | AAC14728.1 | ARO:3002603 | Aminoglycoside resistance | Yes | [116] |
| *APH(6)-Id* | AAC23556.1 | ARO:3002660 | Aminoglycoside resistance | Yes | [121] |
| *ANT(2'')-Ia* | AAC64365.1 | ARO:3000230 | Aminoglycoside resistance | Yes | [122] |
| *arr-2* | AAC64366.1 | ARO:3002847 | Rifamycin resistance | Yes | [123] |
| *tetA(P)* | AAA20116.1 | ARO:3000180 | Efflux pump conferring antibiotic resistance | Yes | [18] |
| *tet(L)* | AAA22851.1 | ARO:3000179 | Efflux pump conferring antibiotic resistance | Yes | [18] |
| *tetS* | AAA25293.1 | ARO:3000192 | Tetracycline resistance | Yes | [124] |
| *lnuA* | AAA26652.1 | ARO:3002835 | Lincosamide resistance | Yes | [125] |
| *ermT* | AAA98096.1 | ARO:3000595 | Macrolide-lincosamide-streptogramin resistance | Yes | [126] |
| *AAC(6')-Ia* | AAA98298.1 | ARO:3002545 | Aminoglycoside resistance | Yes | [127] |
| *cepA* | AAA21532.1 | ARO:3003559 | Beta-lactam resistance | No | N/A |
| *catQ* | AAA23215.1 | ARO:3002687 | Phenicol resistance | No | N/A |
| *vanC* | AAA24786.1 | ARO:3000368 | Glycopeptide resistance | No | N/A |
| *oleC* | AAA26793 | ARO:3003748 | Macrolide resistance | No | N/A |
| *tlrC* | AAA26832.1 | ARO:3002827 | Efflux pump conferring antibiotic resistance | No | N/A |
| *oleB* | AAA50325.1 | ARO:3003036 | Efflux pump conferring antibiotic resistance | No | N/A |
| *mexB* | AAA74437.1 | ARO:3000378 | Efflux pump conferring antibiotic resistance | No | N/A |
| *bcrA* | AAA99504.1 | ARO:3002987 | Bacitracin resistance | No | N/A |
| *tsnR* | AAA99931.1 | ARO:3003060 | Peptide antibiotic resistance | No | N/A |
| *mexC* | AAB41956.1 | ARO:3000800 | Efflux pump conferring antibiotic resistance | No | N/A |
| *mexD* | AAB41957.1 | ARO:3000801 | Efflux pump conferring antibiotic resistance | No | N/A |
| *ceoB* | AAB58161.1 | ARO:3003010 | Efflux pump conferring antibiotic resistance | No | N/A |
| *rosB* | AAC60780.1 | ARO:3003049 | Efflux pump conferring antibiotic resistance | No | N/A |
| *rosA* | AAC60781.1 | ARO:3003048 | Peptide antibiotic resistance | No | N/A |
| *acrB* | AAC73564.1 | ARO:3000216 | Efflux pump conferring antibiotic resistance | No | N/A |
| *kdpE* | AAC73788.1 | ARO:3003841 | Aminoglycoside resistance | No | N/A |
| *msbA* | AAC74000.1 | ARO:3003950 | Efflux pump conferring antibiotic resistance | No | N/A |
| *mdtH* | AAC74149.2 | ARO:3001216 | Efflux pump conferring antibiotic resistance | No | N/A |
| *mdtA* | AAC75135.2 | ARO:3000792 | Efflux pump conferring antibiotic resistance | No | N/A |
| *mdtB* | AAC75136.1 | ARO:3000793 | Efflux pump conferring antibiotic resistance | No | N/A |
| *mdtC* | AAC75137.1 | ARO:3000794 | Efflux pump conferring antibiotic resistance | No | N/A |
| *yojI* | AAC75271.1 | ARO:3003952 | Peptide antibiotic resistance | No | N/A |
| *pmrF* | AAC75314.1 | ARO:3003578 | Peptide antibiotic resistance | No | N/A |
| *evgS* | AAC75429.1 | ARO:3000833 | Efflux pump conferring antibiotic resistance | No | N/A |
| *emrR* | AAC75731.1 | ARO:3000516 | Efflux pump conferring antibiotic resistance | No | N/A |
| *emrB* | AAC75733.1 | ARO:3000074 | Efflux pump conferring antibiotic resistance | No | N/A |
| *bacA* | AAC76093.1 | ARO:3002986 | Peptide antibiotic resistance | No | N/A |
| *acrS* | AAC76296.1 | ARO:3000656 | Efflux pump conferring antibiotic resistance | No | N/A |
| *acrE* | AAC76297.1 | ARO:3000499 | Efflux pump conferring antibiotic resistance | No | N/A |
| *acrF* | AAC76298.1 | ARO:3000502 | Efflux pump conferring antibiotic resistance | No | N/A |
| *mdtF* | AAC76539.1 | ARO:3000796 | Efflux pump conferring antibiotic resistance | No | N/A |
| *vanTC* | AAD22403.1 | ARO:3002970 | Glycopeptide resistance | No | N/A |
| *smeB* | AAD51345.1 | ARO:3003052 | Efflux pump conferring antibiotic resistance | No | N/A |
| *smeC* | AAD51346.1 | ARO:3003053 | Efflux pump conferring antibiotic resistance | No | N/A |
| *smeS* | AAD51347.1 | ARO:3003067 | Efflux pump conferring antibiotic resistance | No | N/A |
| *smeR* | AAD51348.1 | ARO:3003066 | Efflux pump conferring antibiotic resistance | No | N/A |
| *tetT* | AAF01499.1 | ARO:3000193 | Tetracycline resistance | No | N/A |
| *mtrD* | AAF42062.1 | ARO:3000811 | Efflux pump conferring antibiotic resistance | No | N/A |
| *mtrC* | AAF42063.1 | ARO:3000810 | Efflux pump conferring antibiotic resistance | No | N/A |
| *novA* | AAF67494.2 | ARO:3002522 | Aminocoumarin resistance | No | N/A |
| *vanRC* | AAF86641.1 | ARO:3002922 | Glycopeptide resistance | No | N/A |
| *vanSC* | AAF86642.1 | ARO:3002933 | Glycopeptide resistance | No | N/A |
| *triB* | AAG03547.1 | ARO:3003680 | Triclosan resistance | No | N/A |
| *oprM* | AAG03816.1 | ARO:3000379 | Efflux pump conferring antibiotic resistance | No | N/A |
| *mexE* | AAG05881.1 | ARO:3000803 | Efflux pump conferring antibiotic resistance | No | N/A |
| *mexF* | AAG05882.1 | ARO:3000804 | Efflux pump conferring antibiotic resistance | No | N/A |
| *arnA* | AAG06942.1 | ARO:3002985 | Polymyxin resistance | No | N/A |
| *mexK* | AAG07064.1 | ARO:3003693 | Efflux pump conferring antibiotic resistance | No | N/A |
| *mexJ* | AAG07065.1 | ARO:3003692 | Efflux pump conferring antibiotic resistance | No | N/A |
| *mexL* | AAG07066.1 | ARO:3003710 | Efflux pump conferring antibiotic resistance | No | N/A |
| *mexG* | AAG07592.1 | ARO:3000806 | Efflux pump conferring antibiotic resistance | No | N/A |
| *mexH* | AAG07593.1 | ARO:3000807 | Efflux pump conferring antibiotic resistance | No | N/A |
| *mexI* | AAG07594.1 | ARO:3000808 | Efflux pump conferring antibiotic resistance | No | N/A |
| *patB* | AAK76136.1 | ARO:3000025 | Fluoroquinolone resistance | No | N/A |
| *patA* | AAK76137.1 | ARO:3000024 | Fluoroquinolone resistance | No | N/A |
| *pmrA* | AAK99679.1 | ARO:3000822 | Efflux pump conferring antibiotic resistance | No | N/A |
| *adeA* | AAL14439.1 | ARO:3000774 | Efflux pump conferring antibiotic resistance | No | N/A |
| *adeB* | AAL14440.1 | ARO:3000775 | Efflux pump conferring antibiotic resistance | No | N/A |
| *mdsC* | AAL19304.1 | ARO:3000791 | Efflux pump conferring antibiotic resistance | No | N/A |
| *mdsB* | AAL19305.1 | ARO:3000790 | Efflux pump conferring antibiotic resistance | No | N/A |
| *mdsA* | AAL19306.1 | ARO:3000789 | Efflux pump conferring antibiotic resistance | No | N/A |
| *sdiA* | AAL20862.1 | ARO:3000826 | Efflux pump conferring antibiotic resistance | No | N/A |
| *vanE* | AAL27442.1 | ARO:3002907 | Glycopeptide resistance | No | N/A |
| *vanTE* | AAL27444.1 | ARO:3002971 | Glycopeptide resistance | No | N/A |
| *vanRE* | AAL27445.1 | ARO:3002924 | Glycopeptide resistance | No | N/A |
| *vanSE* | AAL27446.1 | ARO:3002935 | Glycopeptide resistance | No | N/A |
| *vanD* | AAM09849.1 | ARO:3000005 | Glycopeptide resistance | No | N/A |
| *vanHD* | AAM09850.1 | ARO:3002944 | Glycopeptide resistance | No | N/A |
| *vanRD* | AAM09851.1 | ARO:3002923 | Glycopeptide resistance | No | N/A |
| *vanXD* | AAM09852.1 | ARO:3003070 | Glycopeptide resistance | No | N/A |
| *mprF* | AAN00989.1 | ARO:3003774 | Peptide antibiotic resistance | No | N/A |
| *murA* | AAN28945 | ARO:3003785 | Fosfomycin resistance | No | N/A |
| *lsaA* | AAO43110.1 | ARO:3000300 | Efflux pump conferring antibiotic resistance | No | N/A |
| *parY* | AAO47226.2 | ARO:3003318 | Aminocoumarin resistance | No | N/A |
| *vanRF* | AAR84672.1 | ARO:3002925 | Glycopeptide resistance | No | N/A |
| *mepA* | AAU95768.1 | ARO:3000026 | Efflux pump conferring antibiotic resistance | No | N/A |
| *macA* | AAV85981.1 | ARO:3000533 | Macrolide resistance | No | N/A |
| *macB* | AAV85982.1 | ARO:3000535 | Macrolide resistance | No | N/A |
| *adeJ* | AAX14802.1 | ARO:3000781 | Efflux pump conferring antibiotic resistance | No | N/A |
| *adeK* | AAX14803.1 | ARO:3000782 | Efflux pump conferring antibiotic resistance | No | N/A |
| *vanUG* | ABA71726.1 | ARO:3004253 | Glycopeptide resistance | No | N/A |
| *vanRG* | ABA71727.1 | ARO:3002926 | Glycopeptide resistance | No | N/A |
| *vanSG* | ABA71728.1 | ARO:3002937 | Glycopeptide resistance | No | N/A |
| *vanWG* | ABA71730.1 | ARO:3002965 | Glycopeptide resistance | No | N/A |
| *vanG* | ABA71731.1 | ARO:3002909 | Glycopeptide resistance | No | N/A |
| *vanXYG* | ABA71732.1 | ARO:3003069 | Glycopeptide resistance | No | N/A |
| *vanTG* | ABA71733.1 | ARO:3002972 | Glycopeptide resistance | No | N/A |
| *arlS* | ABD30512.1 | ARO:3000839 | Efflux pump conferring antibiotic resistance | No | N/A |
| *mprF* | ABG86067.1 | ARO:3003773 | Peptide antibiotic resistance | No | N/A |
| *cmeB* | ABS43151.1 | ARO:3000784 | Efflux pump conferring antibiotic resistance | No | N/A |
| *cmeA* | ABS43901.1 | ARO:3000783 | Efflux pump conferring antibiotic resistance | No | N/A |
| *mdtG* | ABV18113.1 | ARO:3001329 | Efflux pump conferring antibiotic resistance | No | N/A |
| *lmrC* | ABX00624.1 | ARO:3002881 | Lincosamide resistance | No | N/A |
| *vanL* | ABX54687.1 | ARO:3002910 | Glycopeptide resistance | No | N/A |
| *vanTrL* | ABX54690.1 | ARO:3002974 | Glycopeptide resistance | No | N/A |
| *vanRL* | ABX54691.1 | ARO:3002927 | Glycopeptide resistance | No | N/A |
| *tet32* | ACH87088.1 | ARO:3000196 | Tetracycline resistance | No | N/A |
| *adeI* | ACJ41739.1 | ARO:3000780 | Efflux pump conferring antibiotic resistance | No | N/A |
| *abeS* | ACJ59254.1 | ARO:3000768 | Efflux pump conferring antibiotic resistance | No | N/A |
| *tolC* | ACN32294.1 | ARO:3000237 | Efflux pump conferring antibiotic resistance | No | N/A |
| *cfxA6* | ACT97371.1 | ARO:3003097 | Beta-lactam resistance | No | N/A |
| *cipA* | ACX65640.1 | ARO:3003907 | Macrolide- lincosamide-linezolid-phenicol-streptogramin resistance | No | N/A |
| *adeR* | ADM92605.1 | ARO:3000553 | Aminoglycoside resistance | No | N/A |
| *adeS* | ADM92606.1 | ARO:3000549 | Aminoglycoside resistance | No | N/A |
| *hmrM* | ADO96486.1 | ARO:3003953 | Efflux pump conferring antibiotic resistance | No | N/A |
| *ileS* | ADP36409.1 | ARO:3003730 | Mupirocin resistance | No | N/A |
| *lsaC* | AEA37904.1 | ARO:3003112 | Efflux pump conferring antibiotic resistance | No | N/A |
| *mdfA* | AFH35853.1 | ARO:3001328 | Efflux pump conferring antibiotic resistance | No | N/A |
| *salA* | AGN74946 | ARO:3003749 | Lincosamide-streptogramin resistance | No | N/A |
| *adeN* | AGV28567.1 | ARO:3000559 | Efflux pump conferring antibiotic resistance | No | N/A |
| *vanHO* | AHA41499.1 | ARO:3002948 | Glycopeptide resistance | No | N/A |
| *vanO* | AHA41500.1 | ARO:3002913 | Glycopeptide resistance | No | N/A |
| *vanSO* | AHA41504.1 | ARO:3002941 | Glycopeptide resistance | No | N/A |
| *vanRO* | AHA41505.1 | ARO:3002930 | Glycopeptide resistance | No | N/A |
| *rlmA(II)* | AJD73064.1 | ARO:3001301 | Macrolide resistance | No | N/A |
| *adeL* | ALH22601.1 | ARO:3000620 | Efflux pump conferring antibiotic resistance | No | N/A |
| *adeC* | ALX99516.1 | ARO:3003811 | Efflux pump conferring antibiotic resistance | No | N/A |
| *mdtK* | AML99881.1 | ARO:3001327 | Efflux pump conferring antibiotic resistance | No | N/A |
| *gadW* | ANK04027.1 | ARO:3003838 | Efflux pump conferring antibiotic resistance | No | N/A |
| *tetA(58)* | APB03214.1 | ARO:3003980 | Tetracycline resistance | No | N/A |
| *catU* | APB03217.1 | ARO:3003983 | Phenicol resistance | No | N/A |
| *TaeA* | APB03219.1 | ARO:3003986 | Efflux pump conferring antibiotic resistance | No | N/A |
| *arlR* | ATC67679.1 | ARO:3000838 | Efflux pump conferring antibiotic resistance | No | N/A |
| *lmrD* | AYV52072.1 | ARO:3002882 | Lincosamide resistance | No | N/A |
| *emrY* | BAA11237.1 | ARO:3000254 | Efflux pump conferring antibiotic resistance | No | N/A |
| *baeS* | BAA15934.1 | ARO:3000829 | Efflux pump conferring antibiotic resistance | No | N/A |
| *baeR* | BAA15935.1 | ARO:3000828 | Efflux pump conferring antibiotic resistance | No | N/A |
| *acrD* | BAA16344.1 | ARO:3000491 | Efflux pump conferring antibiotic resistance | No | N/A |
| *emrA* | BAA16547.1 | ARO:3000027 | Efflux pump conferring antibiotic resistance | No | N/A |
| *mexY* | BAA34300.1 | ARO:3003033 | Efflux pump conferring antibiotic resistance | No | N/A |
| *AAC(3)-Xa* | BAA78619.1 | ARO:3002544 | Aminoglycoside resistance | No | N/A |
| *H-NS* | BAB35162.1 | ARO:3000676 | Efflux pump conferring antibiotic resistance | No | N/A |
| *evgA* | BAB36671.1 | ARO:3000832 | Efflux pump conferring antibiotic resistance | No | N/A |
| *abeM* | BAD89844.2 | ARO:3000753 | Efflux pump conferring antibiotic resistance | No | N/A |
| *mexN* | BAE06006.1 | ARO:3003705 | Efflux pump conferring antibiotic resistance | No | N/A |
| *gadX* | BAE77778.1 | ARO:3000508 | Efflux pump conferring antibiotic resistance | No | N/A |
| *mdtE* | BAE77781.1 | ARO:3000795 | Efflux pump conferring antibiotic resistance | No | N/A |
| *CRP* | BAE77933.1 | ARO:3000518 | Efflux pump conferring antibiotic resistance | No | N/A |
| *mdtP* | BAE78082.1 | ARO:3003550 | Efflux pump conferring antibiotic resistance | No | N/A |
| *mdtO* | BAE78083.1 | ARO:3003549 | Efflux pump conferring antibiotic resistance | No | N/A |
| *mdtN* | BAE78084.1 | ARO:3003548 | Efflux pump conferring antibiotic resistance | No | N/A |
| *vanWI* | BAE83690.1 | ARO:3003724 | Glycopeptide resistance | No | N/A |
| *vanRI* | BAE85478.1 | ARO:3003728 | Glycopeptide resistance | No | N/A |
| *vanKI* | BAE85481.1 | ARO:3003727 | Glycopeptide resistance | No | N/A |
| *mtrE* | CAA64891.1 | ARO:3000812 | Efflux pump conferring antibiotic resistance | No | N/A |
| *ykkD* | CAB13167.1 | ARO:3003064 | Efflux pump conferring antibiotic resistance | No | N/A |
| *smeE* | CAC14595.1 | ARO:3003056 | Efflux pump conferring antibiotic resistance | No | N/A |
| *smeF* | CAC14596.1 | ARO:3003057 | Efflux pump conferring antibiotic resistance | No | N/A |
| *acrA* | CAC41008.1 | ARO:3004041 | Efflux pump conferring antibiotic resistance | No | N/A |
| *mprF* | CAC99773.1 | ARO:3003770 | Peptide antibiotic resistance | No | N/A |
| *tet36* | CAD55718.1 | ARO:3000197 | tetracycline resistance | No | N/A |
| *cdeA* | CAE00499.1 | ARO:3003835 | Efflux pump conferring antibiotic resistance | No | N/A |
| *amrB* | CAH35802.1 | ARO:3002983 | Efflux pump conferring antibiotic resistance | No | N/A |
| *adeH* | CAJ77855.1 | ARO:3000779 | Efflux pump conferring antibiotic resistance | No | N/A |
| *adeF* | CAJ77856.1 | ARO:3000777 | Efflux pump conferring antibiotic resistance | No | N/A |
| *adeG* | CAJ77857.1 | ARO:3000778 | Efflux pump conferring antibiotic resistance | No | N/A |
| *mprF* | CAX52582.1 | ARO:3003324 | Peptide antibiotic resistance | No | N/A |
| *mtrA* | CCP46065.1 | ARO:3000816 | Efflux pump conferring antibiotic resistance | No | N/A |
| *efrA* | CDO61513.1 | ARO:3003948 | Efflux pump conferring antibiotic resistance | No | N/A |
| *efrB* | CDO61516.1 | ARO:3003949 | Efflux pump conferring antibiotic resistance | No | N/A |
| *lmrB* | KIX81495.1 | ARO:3002813 | Efflux pump conferring antibiotic resistance | No | N/A |
| *CpxR* | SIP52035.1 | ARO:3004054 | Efflux pump conferring antibiotic resistance | No | N/A |
| *abcA* | XP_753111.1 | ARO:3003942 | Efflux pump conferring antibiotic resistance | No | N/A |
| *catB* | AAA73865.1 | ARO:3002674 | Phenicol resistance | No | N/A |
| *acrA* | AAC73565.1 | ARO:3004043 | Efflux pump conferring antibiotic resistance | No | N/A |
| *ugd* | AAC75089.1 | ARO:3003577 | Peptide antibiotic resistance | No | N/A |
| *mdtM* | AAC77293.1 | ARO:3001214 | Efflux pump conferring antibiotic resistance | No | N/A |
| *farA* | AAF40763.1 | ARO:3003961 | Efflux pump conferring antibiotic resistance | No | N/A |
| *farB* | AAF40764.1 | ARO:3003962 | Efflux pump conferring antibiotic resistance | No | N/A |
| *vanXYC* | AAF61331.1 | ARO:3002966 | Glycopeptide resistance | No | N/A |
| *vatF* | AAF63432 | ARO:3003744 | Streptogramin resistance | No | N/A |
| *PmpM* | AAG04750.1 | ARO:3004077 | Efflux pump conferring antibiotic resistance | No | N/A |
| *soxR* | AAG05661.1 | ARO:3004107 | Efflux pump conferring antibiotic resistance | No | N/A |
| *oprN* | AAG05883.1 | ARO:3000805 | Efflux pump conferring antibiotic resistance | No | N/A |
| *opmB* | AAG05913.1 | ARO:3004072 | Efflux pump conferring antibiotic resistance | No | N/A |
| *muxC* | AAG05914 | ARO:3004075 | Efflux pump conferring antibiotic resistance | No | N/A |
| *muxB* | AAG05915.1 | ARO:3004074 | Efflux pump conferring antibiotic resistance | No | N/A |
| *efpA* | CCP45647.1 | ARO:3003955 | Efflux pump conferring antibiotic resistance | No | N/A |
| *norB* | CCQ22388.1 | ARO:3000421 | Efflux pump conferring antibiotic resistance | No | N/A |
| *oprZ* | EGP45230 | ARO:3004142 | Efflux pump conferring antibiotic resistance | No | N/A |
| *axyY* | EGP45231.1 | ARO:3004144 | Efflux pump conferring antibiotic resistance | No | N/A |
| *kpnG* | EHL92831.1 | ARO:3004588 | Efflux pump conferring antibiotic resistance | No | N/A |
| *dfrE* | EOD99669.1 | ARO:3002875 | Diaminopyrimidine resistance | No | N/A |
| *kpnH* | EOU56998.1 | ARO:3004597 | Efflux pump conferring antibiotic resistance | No | N/A |
| *vanI* | KTE89608.1 | ARO:3003723 | Glycopeptide resistance | No | N/A |
| *vmlR* | NP_388442.1 | ARO:3004476 | Macrolide-lincosamide-streptogramin resistance | No | N/A |
| *mprF* | Q8FW76 | ARO:3003772 | Peptide antibiotic resistance | No | N/A |
| *erm(K)* | WP_010896559.1 | ARO:3004643 | Macrolide-lincosamide-streptogramin resistance | No | N/A |
| *otr(A)* | CAA37477.1 | ARO:3002891 | Tetracycline resistance | No | N/A |
| *mupA* | CAA53189 | ARO:3000521 | Mupirocin resistance | No | N/A |
| *APH(3')-Iib* | CAA62365.1 | ARO:3002645 | Aminoglycoside resistance | No | N/A |
| *emrE* | CAA77936.1 | ARO:3004039 | Efflux pump conferring antibiotic resistance | No | N/A |
| *nmcr* | CAA79966.1 | ARO:3003665 | Beta-lactam resistance | No | N/A |
| *tmrB* | CAB12108.2 | ARO:3003059 | Nucleoside antibitoic resistance | No | N/A |
| *aadK* | CAB14620.1 | ARO:3002627 | Aminoglycoside resistance | No | N/A |
| *smeD* | CAC14594.1 | ARO:3003055 | Efflux pump conferring antibiotic resistance | No | N/A |
| *lpeB* | CAH14033.1 | ARO:3004100 | Efflux pump conferring antibiotic resistance | No | N/A |
| *cmlB1* | CAL30186.1 | ARO:3002699 | Efflux pump conferring antibiotic resistance | No | N/A |
| *dfrA26* | CAL48457.1 | ARO:3002857 | Diaminopyrimidine resistance | No | N/A |
| *cfrC* | CAL84423.1 | ARO:3004146 | Macrolide-lincosamide-streptogramin resistance | No | N/A |
| *tet(40)* | CAM12479.1 | ARO:3000567 | Efflux pump conferring antibiotic resistance | No | N/A |
| *ampC1* | CBJ02047.1 | ARO:3004611 | Beta-lactam resistance | No | N/A |
| *opmE* | BAE06009.1 | ARO:3003700 | Efflux pump conferring antibiotic resistance | No | N/A |
| *eptA* | BAE78116.1 | ARO:3003576 | Peptide antibiotic resistance | No | N/A |
| *pgpB* | BAG33043.1 | ARO:3003920 | Peptide antibiotic resistance | No | N/A |
| *efmA* | BAG75524.1 | ARO:3003954 | Efflux pump conferring antibiotic resistance | No | N/A |
| *kpnE* | BAH63251.1 | ARO:3004580 | Efflux pump conferring antibiotic resistance | No | N/A |
| *kpnF* | BAH63252.1 | ARO:3004583 | Efflux pump conferring antibiotic resistance | No | N/A |
| *qacA* | BAJ09383.1 | ARO:3003046 | Efflux pump conferring antibiotic resistance | No | N/A |
| *ampH* | BAJ42218.1 | ARO:3004612 | Beta-lactam resistance | No | N/A |
| *oprA* | BAM10414.1 | ARO:3003039 | Efflux pump conferring antibiotic resistance | No | N/A |
| *ompK37* | CAA09666.1 | ARO:3004122 | Beta-lactam resistance | No | N/A |
| *cpxA* | BAB38260.1 | ARO:3000830 | Efflux pump conferring antibiotic resistance | No | N/A |
| *mgrA* | BAB41874.1 | ARO:3000815 | Efflux pump conferring antibiotic resistance | No | N/A |
| *DHA-1* | BAB43543.1 | ARO:3002132 | Beta-lactam resistance | No | N/A |
| *mepR* | BAB56495.1 | ARO:3000746 | Efflux pump conferring antibiotic resistance | No | N/A |
| *emeA* | BAC11911.1 | ARO:3003551 | Efflux pump conferring antibiotic resistance | No | N/A |
| *rpoB2* | BAD59497.1 | ARO:3000501 | Rifamycin resistance | No | N/A |
| *mexM* | BAE06005.1 | ARO:3003704 | Efflux pump conferring antibiotic resistance | No | N/A |
| *mexP* | BAE06007.1 | ARO:3003698 | Efflux pump conferring antibiotic resistance | No | N/A |
| *mexQ* | BAE06008.1 | ARO:3003699 | Efflux pump conferring antibiotic resistance | No | N/A |
| *mphC* | BAA34540.1 | ARO:3000319 | Macrolide resistance | No | N/A |
| *tetA(60)* | ANZ79240.1 | ARO:3004035 | Efflux pump conferring antibiotic resistance | No | N/A |
| *tetB(60)* | ANZ79241.1 | ARO:3004036 | Efflux pump conferring antibiotic resistance | No | N/A |
| *tetB(48)* | APB03215.1 | ARO:3003981 | Efflux pump conferring antibiotic resistance | No | N/A |
| *LlmA* | APB03216.1 | ARO:3003982 | Lincosamide resistance | No | N/A |
| *rphB* | APB03222.1 | ARO:3003992 | Rifamycin resistance | No | N/A |
| *qepA4* | AQX36338.1 | ARO:3004379 | Efflux pump conferring antibiotic resistance | No | N/A |
| *mecD* | AQX82857.1 | ARO:3004185 | Beta-lactam resistance | No | N/A |
| *catV* | ATL63235.1 | ARO:3004357 | Phenicol resistance | No | N/A |
| *poxtA* | AVI44920.1 | ARO:3004470 | Macrolide-lincosamide-streptogramin resistance | No | N/A |
| *fusF* | AVL76727.1 | ARO:3004663 | Fusidic acid resistance | No | N/A |
| *tcr3* | BAA07390.1 | ARO:3002893 | Tetracycline resistance | No | N/A |
| *rphA* | AIA08936.1 | ARO:3000444 | Rifamycin resistance | No | N/A |
| *ADC-76* | ALA14811.1 | ARO:3003882 | Beta-lactam resistance | No | N/A |
| *bcr-1* | ALV80601.1 | ARO:3003801 | Efflux pump conferring antibiotic resistance | No | N/A |
| *tet(43)* | ACS83748.1 | ARO:3000573 | Efflux pump conferring antibiotic resistance | No | N/A |
| *cblA-1* | ACT97415.1 | ARO:3002999 | Beta-lactam resistance | No | N/A |
| *rbpA* | ADV91011.1 | ARO:3000245 | Rifamycin resistance | No | N/A |
| *PER-7* | AEI54993.1 | ARO:3002369 | Beta-lactam resistance | No | N/A |
| *tet(45)* | AEM62948.1 | ARO:3003196 | Efflux pump conferring antibiotic resistance | No | N/A |
| *lin* | AEO25219.1 | ARO:3004651 | Lincosamide resistance | No | N/A |
| *tetA(46)* | AET10444.1 | ARO:3004032 | Efflux pump conferring antibiotic resistance | No | N/A |
| *tetB(46)* | AET10445.1 | ARO:3004033 | Efflux pump conferring antibiotic resistance | No | N/A |
| *basS* | AEX49906.1 | ARO:3003583 | Peptide antibiotic resistance | No | N/A |
| *mupB* | AEY83581 | ARO:3000510 | mupirocin resistance | No | N/A |
| *ramA* | AFK13828.1 | ARO:3000823 | Efflux pump conferring antibiotic resistance | No | N/A |
| *facT* | AFK80333.1 | ARO:3001313 | Efflux pump conferring antibiotic resistance | No | N/A |
| *rgt1438* | AFO53532.1 | ARO:3002883 | Rifamycin resistance | No | N/A |
| *BRP(MBL)* | AGH88989.1 | ARO:3001205 | Beta-lactam resistance | No | N/A |
| *CMY-104* | AGR82311.1 | ARO:3002116 | Beta-lactam resistance | No | N/A |
| *lnuE* | AGT57825 | ARO:3003762 | Lincosamide resistance | No | N/A |
| *vga(E)* | AHB37625.1 | ARO:3004715 | Streptogramin resistance | No | N/A |
| *mecR1* | ABQ47844.1 | ARO:3000215 | Beta-lactam resistance | No | N/A |
| *blaI* | ABU39978.1 | ARO:3000160 | Beta-lactam resistance | No | N/A |
| *PC1* | ABX30738.1 | ARO:3000621 | Beta-lactam resistance | No | N/A |
| *tet(42)* | ACD35503.1 | ARO:3000572 | Efflux pump conferring antibiotic resistance | No | N/A |
| *mtrR* | ACF30254.1 | ARO:3000817 | Efflux pump conferring antibiotic resistance | No | N/A |
| *abaQ* | ACJ41547.2 | ARO:3004574 | Efflux pump conferring antibiotic resistance | No | N/A |
| *VEB-7* | ACO56763.1 | ARO:3002376 | Beta-lactam resistance | No | N/A |
| *PDC-7* | ACQ82812.1 | ARO:3002506 | Beta-lactam resistance | No | N/A |
| *amvA* | ACQ82816.1 | ARO:3004577 | Efflux pump conferring antibiotic resistance | No | N/A |
| *lmrP* | ABF33001.1 | ARO:3003969 | Efflux pump conferring antibiotic resistance | No | N/A |
| *abaF* | ABO11759.2 | ARO:3004573 | Efflux pump conferring antibiotic resistance | No | N/A |
| *ermR* | AAU93796.1 | ARO:3000594 | Macrolide-lincosamide-streptogramin resistance | No | N/A |
| *CfxA4* | AAV37205.1 | ARO:3003005 | Beta-lactam resistance | No | N/A |
| *tet(38)* | AAV80464.1 | ARO:3000565 | Tetracycline resistance | No | N/A |
| *lmrS* | AAW38464.1 | ARO:3004572 | Efflux pump conferring antibiotic resistance | No | N/A |
| *lnuC* | AAY32951.1 | ARO:3002837 | Lincosamide resistance | No | N/A |
| *mgtA* | ABA28305.2 | ARO:3000462 | Macrolide resistance | No | N/A |
| *oleI* | ABA42118.2 | ARO:3000866 | Macrolide resistance | No | N/A |
| *oleD* | ABA42119.2 | ARO:3000865 | Macrolide resistance | No | N/A |
| *catB9* | AAL68645.1 | ARO:3002681 | Phenicol resistance | No | N/A |
| *dfrC* | AAO04716.1 | ARO:3002865 | Diaminopyrimidine resistance | No | N/A |
| *oqxA* | AAP43109.1 | ARO:3003922 | Fluoroquinolone resistance | No | N/A |
| *omp38* | AAP82271 | ARO:3004123 | Beta-lactam resistance | No | N/A |
| *tet(41)* | AAP93922.1 | ARO:3000569 | Efflux pump conferring antibiotic resistance | No | N/A |
| *qacB* | AAQ10694.1 | ARO:3003047 | Efflux pump conferring antibiotic resistance | No | N/A |
| *OXA-50* | AAQ76277.1 | ARO:3001796 | Beta-lactam resistance | No | N/A |
| *otrC* | AAR96051.1 | ARO:3002894 | Efflux pump conferring antibiotic resistance | No | N/A |
| *norA* | AAS68233.1 | ARO:3000391 | Efflux pump conferring antibiotic resistance | No | N/A |
| *chrB* | AAS79458.1 | ARO:3001302 | Macrolide-lincosamide resistance | No | N/A |
| *mexV* | AAG07762.1 | ARO:3003030 | Efflux pump conferring antibiotic resistance | No | N/A |
| *mexW* | AAG07763.1 | ARO:3003031 | Efflux pump conferring antibiotic resistance | No | N/A |
| *opmH* | AAG08359.1 | ARO:3003682 | Efflux pump conferring antibiotic resistance | No | N/A |
| *emrE* | AAG08375.1 | ARO:3004038 | Efflux pump conferring antibiotic resistance | No | N/A |
| *mphB* | AAG57600.1 | ARO:3000318 | Macrolide resistance | No | N/A |
| *msrC* | AAK01167.1 | ARO:3002819 | Macrolide-lincosamide-streptogramin resistance | No | N/A |
| *tet(35)* | AAK37619.1 | ARO:3000481 | Efflux pump conferring antibiotic resistance | No | N/A |
| *APH(2'')-IIa* | AAK63040.1 | ARO:3002635 | Aminoglycoside resistance | No | N/A |
| *emtA* | AAL02176.1 | ARO:3004669 | Macrolide- lincosamide resistance | No | N/A |
| *fusB* | AAL12234.1 | ARO:3003552 | Fusidic acid resistance | No | N/A |
| *golS* | AAL19308.1 | ARO:3000504 | Efflux pump conferring antibiotic resistance | No | N/A |
| *BlaEC* family class C beta-lactamanse | AAC77110.1 | ARO:3004290 | Beta-lactam resistance | No | N/A |
| *otr(B)* | AAD04032.1 | ARO:3002892 | Tetracycline resistance | No | N/A |
| *mef(En2)* | AAF74725.1 | ARO:3004659 | Macrolide resistance | No | N/A |
| *ermB* | AAF86219.1 | ARO:3000375 | Macrolide-lincosamide-streptogramin resistance | No | N/A |
| *triC* | AAG03548.1 | ARO:3003681 | Triclosan resistance | No | N/A |
| *mexA* | AAG03814.1 | ARO:3000377 | Efflux pump conferring antibiotic resistance | No | N/A |
| *catB7* | AAG04095.1 | ARO:3002679 | Phenicol resistance | No | N/A |
| *fosA* | AAG04518.1 | ARO:3000149 | Fosfomycin resistance | No | N/A |
| *muxA* | AAG05916.1 | ARO:3004073 | Efflux pump conferring antibiotic resistance | No | N/A |
| *opmD* | AAG07595.1 | ARO:3000809 | Efflux pump conferring antibiotic resistance | No | N/A |
| *ermX* | AAA98484.1 | ARO:3000596 | Macrolide-lincosamide-streptogramin resistance | No | N/A |
| *cmlv* | AAB36568.1 | ARO:3002700 | Phenicol resistance | No | N/A |
| *oprJ* | AAB41958.1 | ARO:3000802 | Efflux pump conferring antibiotic resistance | No | N/A |
| *AAC(6')-Ii* | AAB63533.1 | ARO:3002556 | Aminoglycoside resistance | No | N/A |
| *tet(V)* | AAB84282.1 | ARO:3000181 | Tetracycline resistance | No | N/A |
| *blt* | AAC36944.1 | ARO:3003006 | Efflux pump conferring antibiotic resistance | No | N/A |
| *cmrA* | AAC45805.1 | ARO:3002702 | Phenicol resistance | No | N/A |
| *rpoB* mutants | A1A317 | ARO:3004480 | Rifamycin resistance | No | N/A |
| *catA4* | AAA25655.1 | ARO:3004657 | Phenicol resistance | No | N/A |
| *aadS* | AAA27459.1 | ARO:3004683 | Aminoglycoside resistance | No | N/A |
| *ermF* | AAA88675.1 | ARO:3000498 | Macrolide-lincosamide-streptogramin resistance | No | N/A |
