# Supplementary material for: Investigating potential transmission of antimicrobial resistance in an open-plan hospital ward: a cross-sectional metagenomic study of resistome dispersion in a lower middle-income setting
Source: Antimicrob Resist Infect Control. 2021 Mar 18;10:56. doi: 10.1186/s13756-021-00915-w (PMC7977308; doi:10.1186/s13756-021-00915-w)
Supplement: Supplementary file 5 — Additional file 5: Table S4. Antibiotic use according to class of antibiotic during study period and annual hospital use 2017-2018. [file 13756_2021_915_MOESM5_ESM.docx]

**Table S4**: Antibiotic use according to class of antibiotic during study period and annual hospital use 2017-2018.

| **Class of antibiotic** | **% of usage** |
| --- | --- |
| **During study period** |  |
| Cephalosporins | 23·7 |
| Anti-TB agents (first and second line) | 20·3 |
| Fluoroquinolones | 16·9 |
| Penicillins and combination penicillin | 13·6 |
| Combination cephalosporins | 8·5 |
| Nitroimidazoles | 5·1 |
| Aminoglycosides | 3·4 |
| Carbapenems | 3·4 |
| Rifaximin | 3·4 |
| Macrolides | 1·7 |
| **Annual hospital use** |  |
| Penicillins and combination penicillins | 35 |
| Cephalosporins | 25 |
| Co-trimoxazole | 12 |
| Anti-TB agents (first and second line) | 10 |
| Fluoroquinolones | 6 |
| Macrolides | 3 |
| Rifamycins | 3 |
| Lincomycins | 2·4 |
| Aminoglycosides | 2 |
| Carbapenems | 1 |
| Glycopeptides | 0·3 |
| Others | 0·3 |
