# Supplementary material for: Investigating potential transmission of antimicrobial resistance in an open-plan hospital ward: a cross-sectional metagenomic study of resistome dispersion in a lower middle-income setting
Source: Antimicrob Resist Infect Control. 2021 Mar 18;10:56. doi: 10.1186/s13756-021-00915-w (PMC7977308; doi:10.1186/s13756-021-00915-w)
Supplement: Supplementary file 6 — Additional file 6: Table S5. Significant correlations between detected AMR genes and bacterial taxa (n=24 patients) [file 13756_2021_915_MOESM6_ESM.docx]

**Table S5:** Significant correlations between detected AMR genes and bacterial taxa (n=24 patients).

| **Gene** | **Antibiotic resistance** | **Gene Regulator** | **Phylum: Taxa** | **Spearman’s r** | **P value** |
| --- | --- | --- | --- | --- | --- |
| *ACI-1* ^†^ | Beta-lactam resistance | No | F: *Ruminococcaceae UCG-014* * | 0.83 | <0.01 |
| *basS* | Peptide antibiotic resistance | No | F: *Erysipelotrichaceae UCG-003 ** | 0.74 | <0.01 |
| *blaR1* ^†^ | Beta-lactam resistance | Yes | F: *Ruminiclostridium 6* *  F: *Megamonas* *  F: *Ruminococcus 1* *  A: *Enterorhabdus* * | 0.65  0.80  0.84  0.84 | 0.03  <0.01  <0.01  <0.01 |
| *catB10* | Phenicol resistance | No | F: *Ruminococcaceae NK4A214 group ***  F: *Ruminiclostridium 6 *** | 0.76  0.79 | <0.01  <0.01 |
| *catV* | Phenicol resistance | No | F: *Subdoligranulum **  F: *Megamonas **  A: *Enterorhabdus ***  F: *Ruminococcus 1 ** | 0.65  0.67  0.74  0.74 | 0.04  0.03  <0.01  <0.01 |
| *cblA-1* | Beta-lactam resistance | No | A: *Olsenella *** | 0.81 | <0.01 |
| *clbA* | Macrolide-lincosamide-linezolid-phenicol-streptogramin resistance | No | B: *Prevotella 7 ** | 0.68 | 0.02 |
| *dfrA1* | Diaminopyrimidine resistance | No | A: *Olsenella ***  F: *Clostridioides ***  F: *Moryella *** | 0.66  0.74  0.70 | 0.03  <0.01  0.13 |
| *dfrA15* | Diaminopyrimidine resistance | No | A: *Senegalimassilia *** | 0.65 | 0.04 |
| *farA* | Efflux pump conferring antibiotic resistance | No | P: *Desulfovibrio **  F: *Peptococcus ***  F: *Lachnospiraceae NK4A136 group ***  P: Burkholderiaceae_unclassified ***  F: *Ruminococcaceae UCG-004 ***  B: *Barnesiella **  F: *Oscillibacter *** | 0.64  0.64  0.76  0.78  0.83  0.79  0.83 | 0.04  0.04  <0.01  <0.01  <0.01  <0.01  <0.01 |
| *fexA* ^†^ | Efflux pump conferring antibiotic resistance | No | F: *Tyzzerella 4 **  F: *[Ruminococcus] torques group ** | 0.67  0.66 | 0.02  0.02 |
| *lnuA* ^†^ | Lincosamide resistance | No | F: *Weissella **  F: *Ruminococcaceae.NK4A214 group ** | 0.71  0.68 | <0.01  0.02 |
| *mcr-1* | Peptide antibiotic resistance | No | F: *Clostridioides *** | 0.83 | <0.01 |
| *mef(B)* ^†^ | Macrolide resistance | No | F: *Ruminococcaceae NK4A214 group ** | 0.65 | 0.03 |
| *mexL* | Efflux pump conferring antibiotic resistance |  | F: *Ruminococcaceae UCG-002* ** | 0.66 | 0.03 |
| *nmcr* | Beta-lactam resistance | Yes | F: *Peptostreptococcus ** | 0.73 | <0.01 |
| *opmE* | Efflux pump conferring antibiotic resistance | No | B: *Alloprevotella **  F: *Lachnospiraceae UCG-004 ** | 0.75  0.78 | <0.01  <0.01 |
| *oprA* | Efflux pump conferring antibiotic resistance | No | F: *Dielma ** | 0.86 | <0.01 |
| *optrA* ^†^ | Mactrolide-streptogramin-lincosamide resistance | No | F: *Catenibacterium ** | 0.65 | 0.04 |
| *OXA-181* | Beta-lactam resistance | No | F: *Dielma ** | 0.68 | 0.02 |
| *pgpB* | Peptide antibiotic resistance | No | B: *Prevotella 9 ** | 0.64 | 0.04 |
| *poxtA* | Macrolide-lincosamide-streptogramin resistance | No | A: *Corynebacterium ** | -0.64 | 0.04 |
| *qacB* | Efflux pump conferring antibiotic resistance | No | F: *Dielma ** | 0.74 | <0.01 |
| *salA* | Lincosamide-streptogramin resistance | No | F: *Hungatella **  F: Family XIII_unclassified *** | 0.68  0.75 | 0.02  <0.01 |
| *sat1* ^†^ | Nucleoside antibiotic resistance | No | F: *Clostridioides **  F: *Moryella ** | 0.81  0.64 | <0.01  0.04 |
| *smeD* | Efflux pump conferring antibiotic resistance | No | F: *Megasphaera *** | 0.69 | 0.02 |
| *sul3* | Sulfonamide resistance | No | F: *Erysipelatoclostridium *** | 0.64 | 0.04 |
| *tetA(46)* | Efflux pump conferring antibiotic resistance | No | A: *Collinsella ** | 0.68 | 0.02 |
| *tetA(60)* | Efflux pump conferring antibiotic resistance | No | F: *Mogibacterium ** | 0.64 | 0.04 |
| *tetA(P)* ^†^ | Efflux pump conferring antibiotic resistance | No | F: *Murdochiella ** | 0.77 | <0.01 |
| *tetB(48)* | Efflux pump conferring antibiotic resistance | No | F: *Ruminococcaceae NK4A214 group ** | 0.65 | 0.03 |
| *tet(D)* | Efflux pump conferring antibiotic resistance | No | F: *[Ruminococcus] torques group ***  F: *Moryella *** | 0.70  0.64 | 0.01  0.04 |
| *tet32* | Tetracycline resistance | No | F: *Subdoligranulum ** | 0.64 | 0.04 |
| *tlrC* | Efflux pump conferring antibiotic resistance | No | B: *Prevotella 7 ***  F: Family XIII_unclassified *** | 0.71  0.68 | 0.01  0.02 |
| *tmrB* | Nucleoside antibiotic resistance | No | F: Peptostreptococcaceae_unclassified *** | 0.67 | 0.02 |
| *vanE* | Glycopeptide resistance | No | B: *Prevotella 9 *** | 0.65 | 0.03 |
| *vanHA* | Glycopeptide resistance | No | F: *Catenibacterium ** | 0.81 | <0.01 |
| *vanO* | Glycopeptide resistance | No | F: *Megamonas ** | 0.79 | <0.01 |
| *vanRD* | Glycopeptide resistance | No | F: *Subdoligranulum ** | 0.76 | <0.01 |
| *vanSA* | Glycopeptide resistance | No | A: Corynebacteriaceae_unclassified *** | 0.64 | 0.04 |
| *vanSM* | Glycopeptide resistance | No | F: *Megasphaera ** | 0.65 | 0.04 |
| *vanSN* | Glycopeptide resistance | No | F: *Catenibacterium **  F: *Ruminococcaceae NK4A214 group **  F: *Ruminiclostridium 6 ** | 0.67  0.68  0.68 | 0.03  0.02  0.02 |
| *vanWB* | Glycopeptide resistance | No | F: *Megamonas ** | 0.67 | 0.02 |
| *vanWG* | Glycopeptide resistance | No | F: *Lachnospiraceae NK4A136 group **  F: *Megamonas ** | 0.64  0.74 | 0.04  <0.01 |
| *vgaC* ^†^ | Mactrolide-streptogramin-lincosamide resistance | No | F: *Ruminococcus 1 **  A: *Enterorhabdus ** | 0.65  0.65 | 0.04  0.04 |
| *vgaE* ^†^ | Mactrolide-streptogramin-lincosamide resistance | No | P: *Desulfovibrio ***  F: *Ruminococcus **  F: *Ruminiclostridium 6 **  B: *Odoribacter ***  F: *Solobacterium ** | 0.64  0.83  0.64  0.79  0.84 | 0.04  <0.01  0.04  <0.01  <0.01 |

Phylum: F- Firmicutes; A-Actinobacteria; B- Bacteroidetes; P- Proteobacteria

* Possible association/No data with resistance gene

** No association with resistance gene

^†^ Evidence of gene mobility
