# Supplementary material for: Investigating potential transmission of antimicrobial resistance in an open-plan hospital ward: a cross-sectional metagenomic study of resistome dispersion in a lower middle-income setting
Source: Antimicrob Resist Infect Control. 2021 Mar 18;10:56. doi: 10.1186/s13756-021-00915-w (PMC7977308; doi:10.1186/s13756-021-00915-w)
Supplement: Supplementary file 7 — Additional file 7: Table S6. Proportion of genes detected in shotgun metagenomic sequencing and qPCR analysis. [file 13756_2021_915_MOESM7_ESM.docx]

**Table S6:** Proportion of genes detected in shotgun metagenomic sequencing and qPCR analysis.

| **Gene** | **Proportion detected in shotgun metagenomics**  **(n=24)** | **Proportion detected in qPCR**  **(n=59)** |
| --- | --- | --- |
| *OXA-1* | 25·0% | 24·0% |
| *CTX-M-14* | 16·7% | 25·4% |
| *CMY-2* | 45·8% | 31·0% |
| *mcr-1.0* | 12·5% | 5·1% |
| *NDM-7* | 41·7% | 30·5% |
| *drfA14* | 29·2% | 28·8% |
| *catB3* | 16·7% | 22·0% |
| *fusB* | 16·7% | 40·7% |
| *rmtB* | 16·7% | 15·3% |
