# Supplementary material for: Investigating potential transmission of antimicrobial resistance in an open-plan hospital ward: a cross-sectional metagenomic study of resistome dispersion in a lower middle-income setting
Source: Antimicrob Resist Infect Control. 2021 Mar 18;10:56. doi: 10.1186/s13756-021-00915-w (PMC7977308; doi:10.1186/s13756-021-00915-w)
Supplement: Supplementary file 8 — Additional file 8: Table S7. Distribution of resistance genes according to patient location on the ward. [file 13756_2021_915_MOESM8_ESM.docx]

**Table S7:** Distribution of resistance genes according to patient location on the ward.

| **Location** | ***OXA-1*** | ***NDM-7*** | ***mcr-1·0*** | ***CTX-M-14*** | ***CMY-2*** | ***dfrA14*** | ***catB3*** | ***fusB*** | ***rmtB*** |
| --- | --- | --- | --- | --- | --- | --- | --- | --- | --- |
| Private rooms | 0 | 0 | 0 | 0 | 0 | 50·0% | 0 | 0 | 0 |
| Bay 1 | 11·0% | 0 | 0 | 22·2% | 11·0% | 22·2% | 11·0% | 66·7% | 0 |
| Bay 2 | 0 | 37·5% | 0 | 25·0% | 37·5% | 0 | 0 | 25·0% | 12·5% |
| HDU | 0 | 25·0% | 25·0% | 0 | 25·0% | 0 | 0 | 0 | 25·0% |
| Bay 4 | 12·5% | 0 | 0 | 37·5% | 12·5% | 0 | 0 | 25·0% | 0 |
| Bay 5 | 50·0% | 75·0% | 25·0% | 50·0% | 100·0% | 50·0% | 50·0% | 75·0% | 25·0% |
| Bay 6 | 28·6% | 28·6% | 14·3% | 14·3% | 14·3% | 42·9% | 28·6% | 57·1% | 14·3% |
| Bay 7 | 66·7% | 50·0% | 0 | 33·3% | 33·3% | 50·0% | 66·7% | 83·3% | 50·0% |
| Bay 8 | 50·0% | 75·0% | 0 | 0 | 75·0% | 50·0% | 50·0% | 50·0% | 25·0% |
| Patients within corridor | 28·6% | 42·9% | 0 | 42·9% | 28·6% | 57·1% | 28·6% | 0 | 14·3% |
