# Supplementary material for: Investigating potential transmission of antimicrobial resistance in an open-plan hospital ward: a cross-sectional metagenomic study of resistome dispersion in a lower middle-income setting
Source: Antimicrob Resist Infect Control. 2021 Mar 18;10:56. doi: 10.1186/s13756-021-00915-w (PMC7977308; doi:10.1186/s13756-021-00915-w)
Supplement: Supplementary file 9 — Additional file 9: Table S8. Univariate analysis of clinical variables and resistance genes. [file 13756_2021_915_MOESM9_ESM.docx]

**Table S8:** Univariate analysis of clinical variables and resistance genes.

| **Genes** | **Length of hospital stay^+^** | **Use of antibiotics^+^** | **Use of gram-negative antibiotics^+^** | **Patients with TB^+^** | **Patients with bacterial sepsis^+^** | **Presence of bacterial sepsis and MDR TB^+^** |
| --- | --- | --- | --- | --- | --- | --- |
| *OXA-1* | 0·25 | 0·60 | 0·43 | 0·02 | 0·29 | 0·28 |
| *NDM-7* | 0·02 | 0·08 | 0·40 | 0·06 | 0·50 | 0·27 |
| *catB3* | 0·21 | 0·11 | 0·85 | <0·01 | 0·84 | 0·88 |
| *dfrA14* | 0·28 | 0·31 | 0·20 | <0·01 | 0·18 | 0·39 |
| *rmtB* | 0·81 | 0·27 | 0·62 | 0·06 | 0·65 | 0·47 |
| *fusB* | 0·62 | 0·78 | 0·90 | 0·87 | 0·50 | 0·46 |
| *CTX-M-14* | 0·94 | 0·15 | 0·19 | 0·84 | 0·34 | 0·34 |
| *CMY-2* | 0·04 | 0·03 | 0·19 | 0·51 | 0·08 | 0·10 |
| *mcr-1·0* | 0·35 | 0·96 | 0·95 | 0·96 | 0·93 | 0·94 |

**^+^**Adjusted for age

TB: tuberculosis; MDR TB: multi-drug resistant tuberculosis
