# Supplementary material for: Investigating potential transmission of antimicrobial resistance in an open-plan hospital ward: a cross-sectional metagenomic study of resistome dispersion in a lower middle-income setting
Source: Antimicrob Resist Infect Control. 2021 Mar 18;10:56. doi: 10.1186/s13756-021-00915-w (PMC7977308; doi:10.1186/s13756-021-00915-w)
Supplement: Supplementary file 10 — Additional file 10: Table S9. Multivariate regression analysis of clinical variables and resistance genes. [file 13756_2021_915_MOESM10_ESM.docx]

**Table S9:** Multivariate regression analysis of clinical variables and resistance genes.

| **Genes** | **Length of hospital stay^+^** | **Patients with TB^+^** | **Patients with bacterial sepsis^+^** |
| --- | --- | --- | --- |
| *OXA-1* | 0·60 | 0·03 | 0·79 |
| *NDM-7* | 0·04 | 0·13 | 0·13 |
| *catB3* | 0·45 | <0·01 | 0·37 |
| *dfrA14* | 0·72 | 0·02 | 0·58 |
| *rmtB* | 0·89 | 0·03 | 0·27 |
| *fusB* | 0·60 | 0·61 | 0·47 |
| *CTX-M-14* | 0·91 | 0·64 | 0·29 |
| *CMY-2* | 0·03 | 0·59 | 0·03 |
| *mcr-1·0* | 0·41 | 0·61 | 0·36 |

**^+^**Adjusted for age, length of stay, patients with TB and presence of bacterial sepsis

TB: tuberculosis
