# Supplementary material for: Investigating potential transmission of antimicrobial resistance in an open-plan hospital ward: a cross-sectional metagenomic study of resistome dispersion in a lower middle-income setting
Source: Antimicrob Resist Infect Control. 2021 Mar 18;10:56. doi: 10.1186/s13756-021-00915-w (PMC7977308; doi:10.1186/s13756-021-00915-w)
Supplement: Supplementary file 11 — Additional file 11: Table S10. Correlation between relative abundance of specific taxa and absolute abundance of associated AMR genes. [file 13756_2021_915_MOESM11_ESM.docx]

**Table S10:** Correlation between relative abundance of specific taxa and absolute abundance of associated AMR genes.

| **Taxa** | **Gene** | **Spearman r** | **P value** |
| --- | --- | --- | --- |
| *Escherichia-Shigella* | *OXA-1*  *NDM-7*  *dfrA14*  *rmtB*  *catB3* | 0.43  0.47  0.36  0.13  0.47 | <0·01  <0·01  <0·01  0.33  <0·01 |
| *Pseudomonas sp.* | *OXA-1*  *catB3* | -0.18  -0.17 | 0.20  0.20 |
| *Acinetobacter sp.* | *rmtB*  *catB3* | -0.02  -0.09 | 0.86  0.49 |
| *Staphylococcus sp.* | *fusB* | 0.23 | 0.07 |
| *Morganella sp.* | *OXA-1*  *dfrA14*  *rmtB*  *catB3* | 0.19  -0.01  0.13  0.04 | 0.15  0.98  0.31  0.76 |
| *Proteus sp.* | *OXA-1*  *dfrA14*  *rmtB*  *catB3* | -0.04  0.18  0.05  0.18 | 0.78  0.16  0.72  0.17 |
