# Supplementary figures and images for: Investigating potential transmission of antimicrobial resistance in an open-plan hospital ward: a cross-sectional metagenomic study of resistome dispersion in a lower middle-income setting
Source: Antimicrob Resist Infect Control. 2021 Mar 18;10:56. doi: 10.1186/s13756-021-00915-w (PMC7977308; doi:10.1186/s13756-021-00915-w)

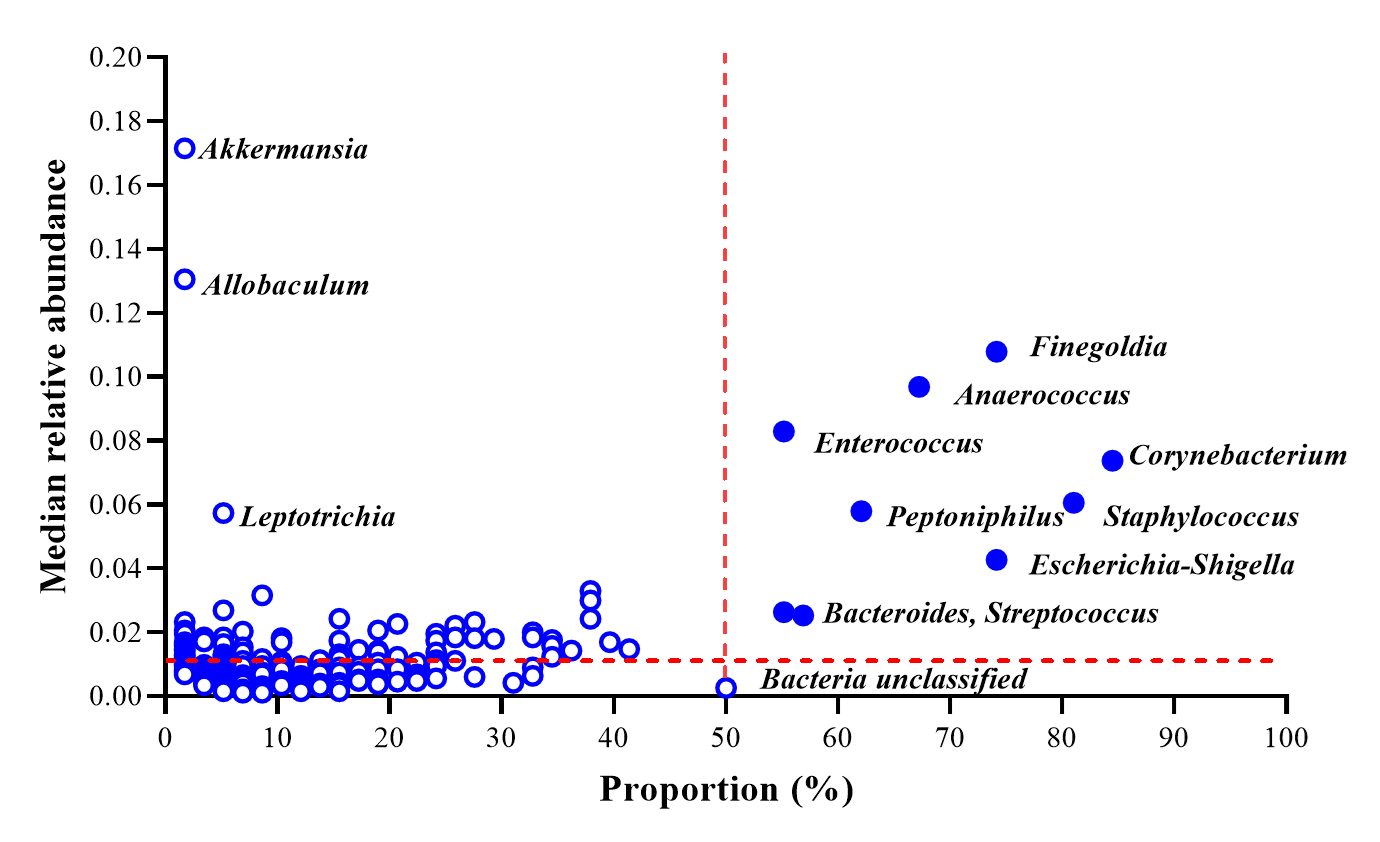

Supplement: Supplementary file 12 — Additional file 12: Figure S1. Identification of core microbiota among study population (>50% population, median>0.01). [file 13756_2021_915_MOESM12_ESM.tif]

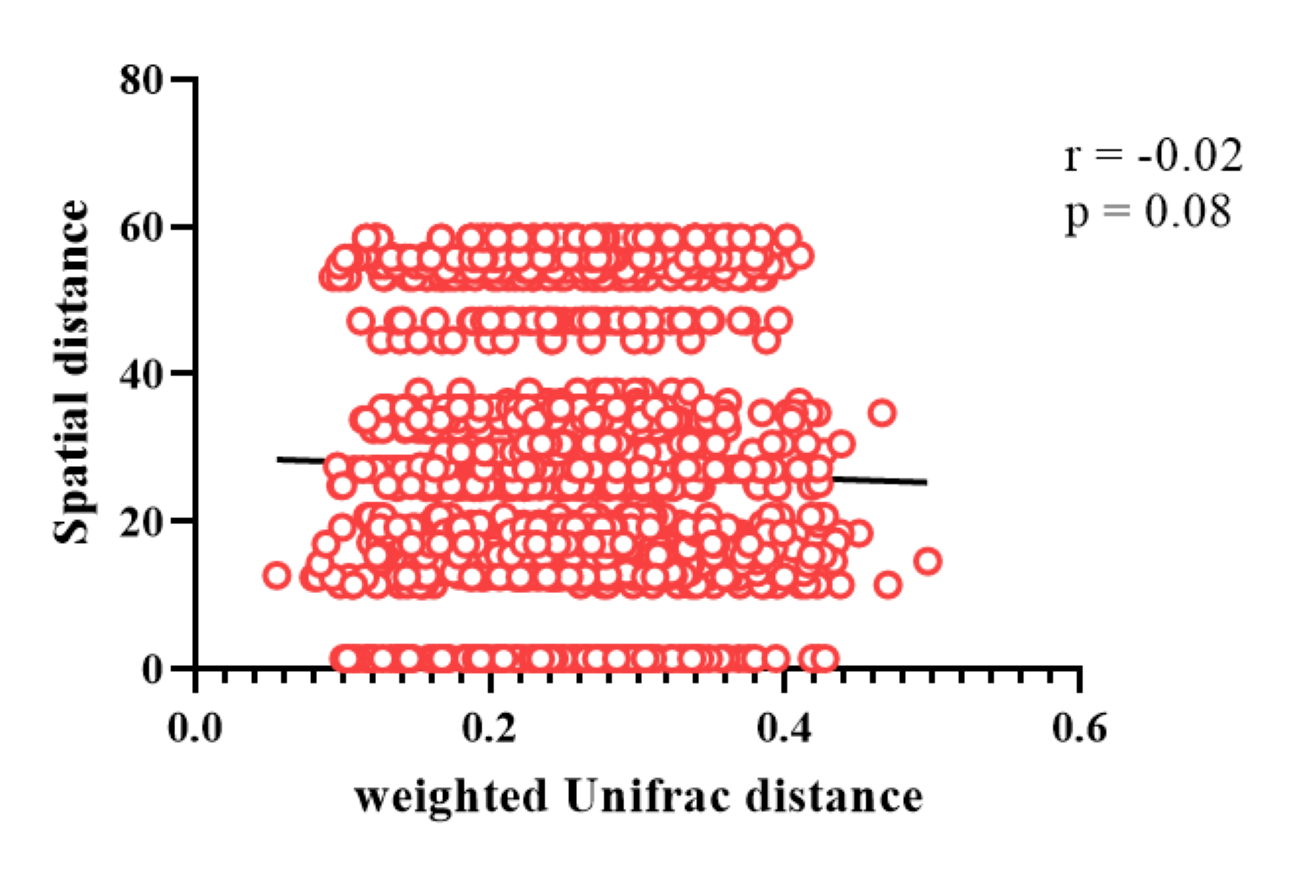

Supplement: Supplementary file 13 — Additional file 13: Figure S2. Spearman’s correlation of spatial distance between individuals located on the ward (spatial distance) and compositional similarity distance between individual’s microbiota (weighted UNIFRAC distance). [file 13756_2021_915_MOESM13_ESM.tif]

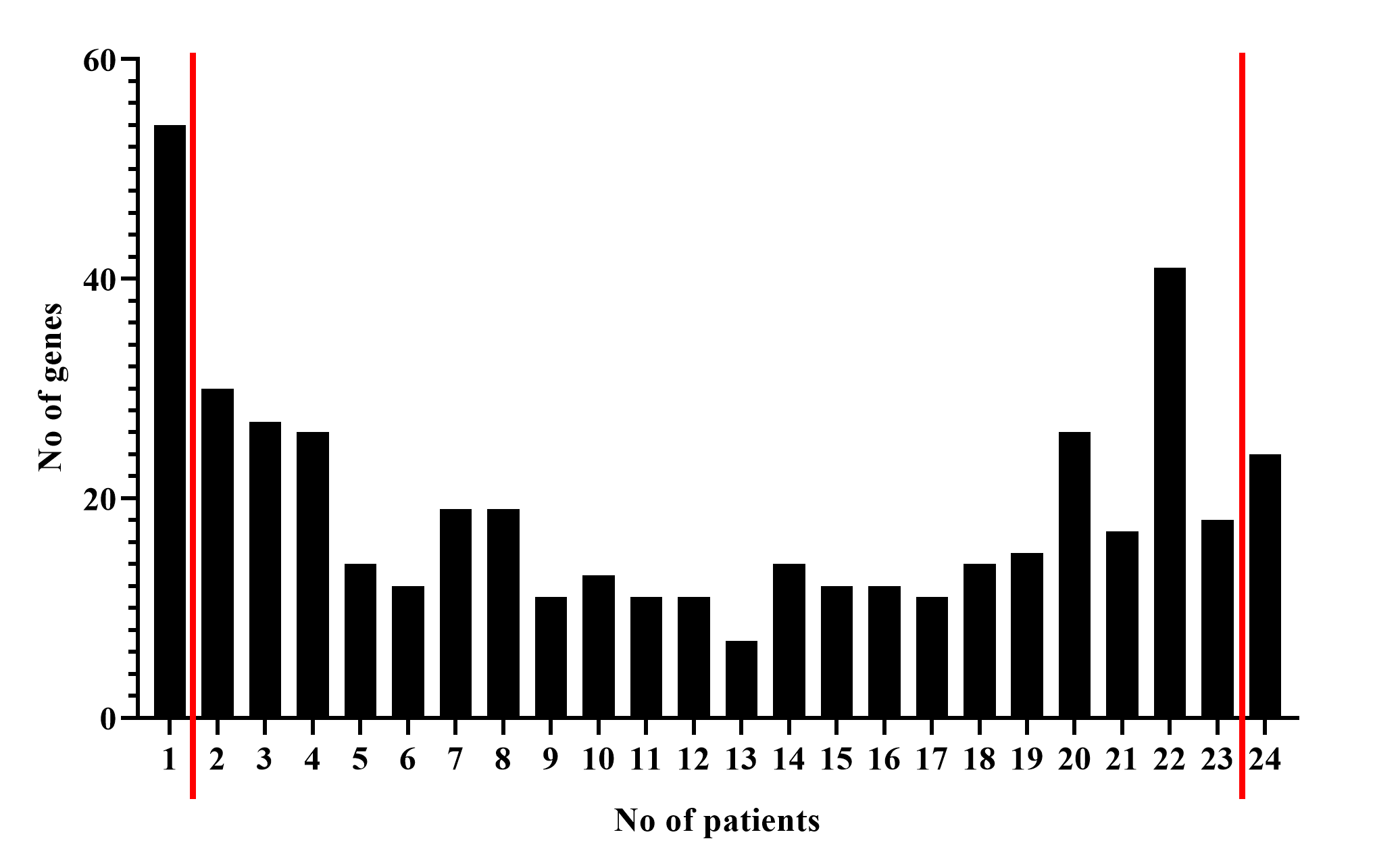

Supplement: Supplementary file 14 — Additional file 14: Figure S3. Distribution of detected resistance genes within patient cohort. The number of patients (of a total of 24) in whom a specific number of resistance were detected is shown. Genes that detected in all patients, or in only a single individual only, were excluded from analysis of potential inter-patient transmission. [file 13756_2021_915_MOESM14_ESM.tif]

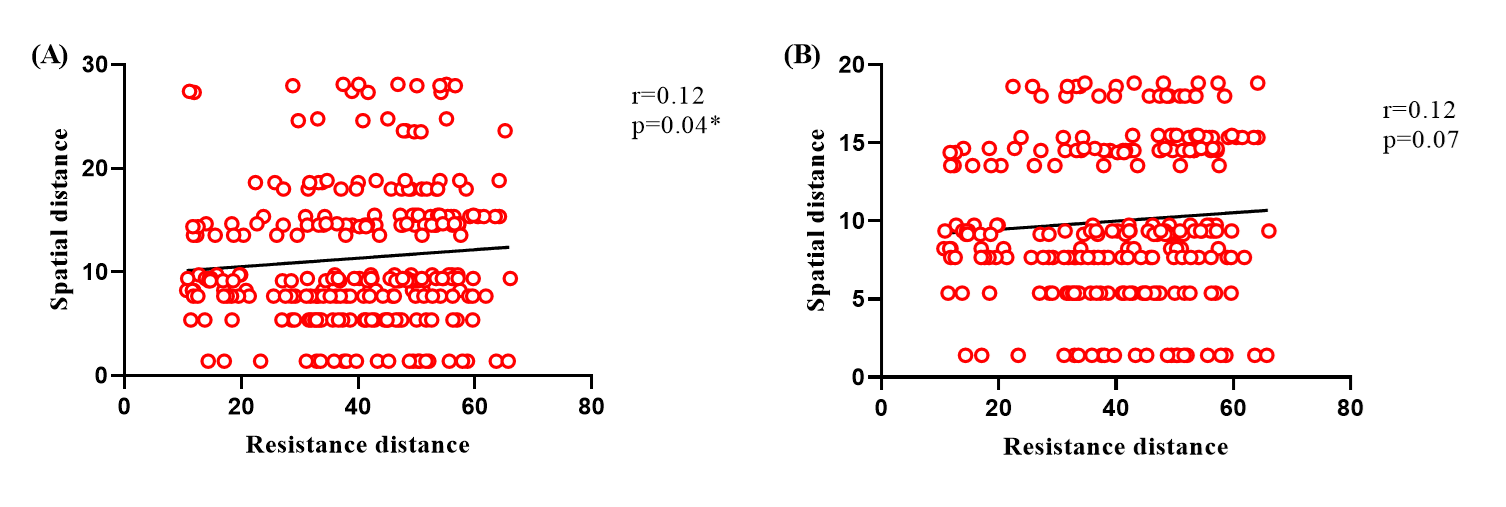

Supplement: Supplementary file 15 — Additional file 15: Figure S4. Spearman’s correlation of spatial distance between individuals located on the ward (spatial distance) and resistance gene presence/absence similarity (resistance distance). (A) Resistance genes present in patients located within the bay and corridor (B) resistance genes present in patients located within the bay. [file 13756_2021_915_MOESM15_ESM.tif]

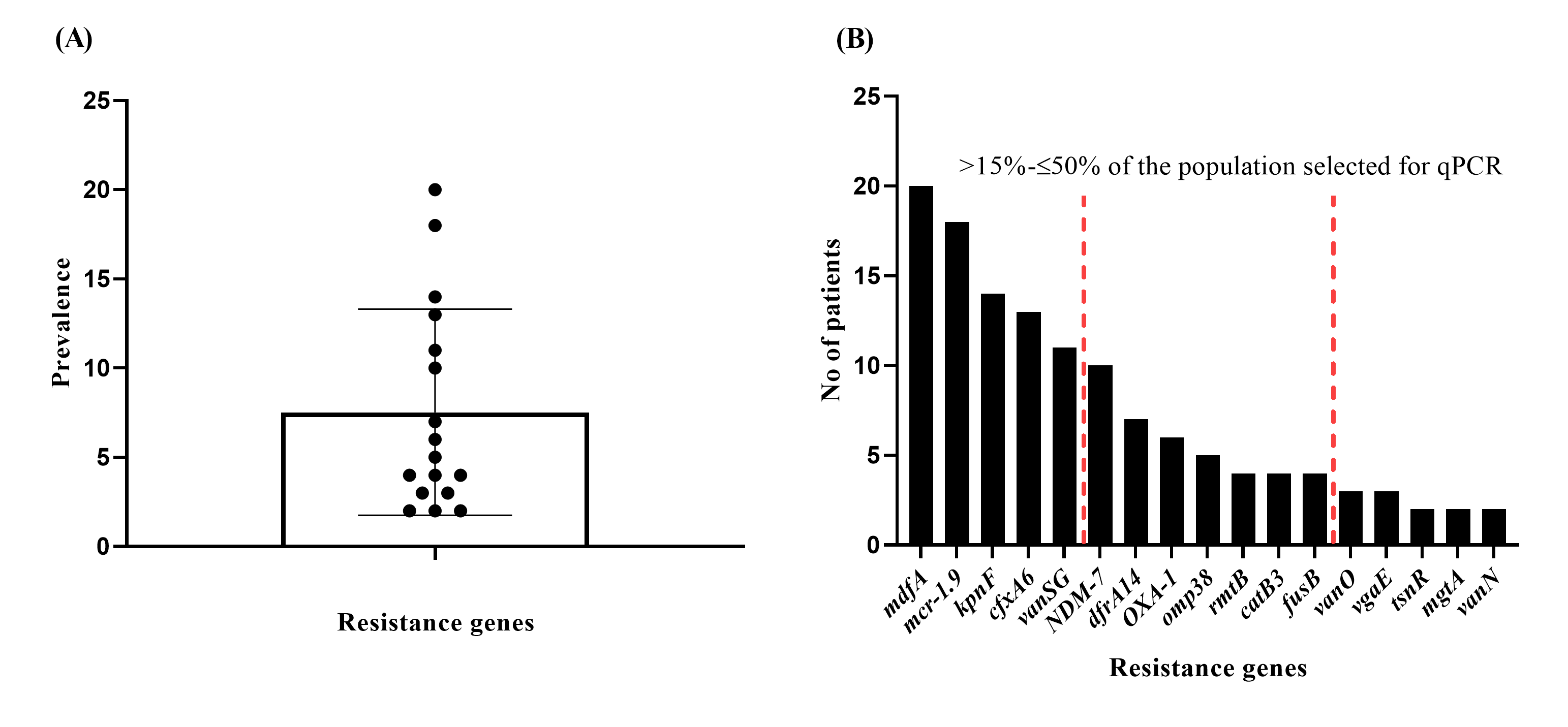

Supplement: Supplementary file 16 — Additional file 16: Figure S5. (A) Overall distribution of resistance genes identified through DISTLM according to their prevalence (B) Prevalence of individual genes as detected by metagenomic analysis. Genes present in >15% and <50% were selected for validation by quantitative PCR. [file 13756_2021_915_MOESM16_ESM.tif]

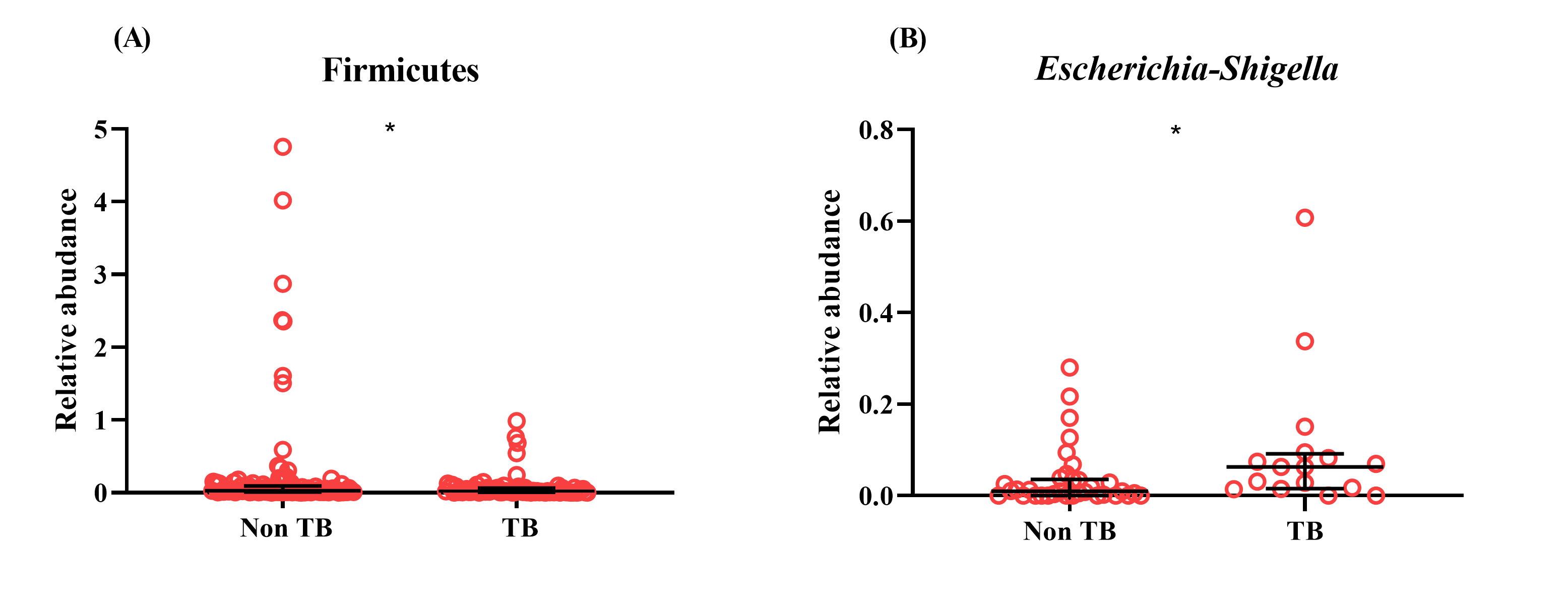

Supplement: Supplementary file 17 — Additional file 17: Figure S6. (A) Levels of Firmicutes between patients with and without TB (B) Levels of Escherichia-Shigella between patients with and without TB. [file 13756_2021_915_MOESM17_ESM.tif]
